# Identification and characterization of the glucose dual-affinity transport system in *Neurospora crassa*: pleiotropic roles in nutrient transport, signaling, and carbon catabolite repression

**DOI:** 10.1186/s13068-017-0705-4

**Published:** 2017-01-19

**Authors:** Bang Wang, Jingen Li, Jingfang Gao, Pengli Cai, Xiaoyun Han, Chaoguang Tian

**Affiliations:** 1 Key Laboratory of Systems Microbial Biotechnology, Tianjin Institute of Industrial Biotechnology, Chinese Academy of Sciences, Tianjin, 300308 China; 2 University of Chinese Academy of Sciences, Beijing, 100049 China; 3 School of Ophthalmology and Optometry, Eye Hospital, State Key Laboratory Cultivation Base and Key Laboratory of Vision Science, Ministry of Health and Zhejiang Provincial Key Laboratory of Ophthalmology and Optometry, Wenzhou Medical University, Wenzhou, 325027 China; 4 School of Life Sciences, Heilongjiang University, Harbin, 150080 Heilongjiang China

**Keywords:** Glucose transporter, Dual-affinity, GLT-1, HGT-1/-2, Cellulase, Signaling

## Abstract

**Background:**

The glucose dual-affinity transport system (low- and high-affinity) is a conserved strategy used by microorganisms to cope with natural fluctuations in nutrient availability in the environment. The glucose-sensing and uptake processes are believed to be tightly associated with cellulase expression regulation in cellulolytic fungi. However, both the identities and functions of the major molecular components of this evolutionarily conserved system in filamentous fungi remain elusive. Here, we systematically identified and characterized the components of the glucose dual-affinity transport system in the model fungus *Neurospora crassa*.

**Results:**

Using RNA sequencing coupled with functional transport analyses, we assigned GLT-1 (*K*_m_ = 18.42 ± 3.38 mM) and HGT-1/-2 (*K*_m_ = 16.13 ± 0.95 and 98.97 ± 22.02 µM) to the low- and high-affinity glucose transport systems, respectively. The high-affinity transporters *hgt*-*1/*-*2* complemented a moderate growth defect under high glucose when *glt*-*1* was deleted. Simultaneous deletion of *hgt*-*1/*-*2* led to extensive derepression of genes for plant cell wall deconstruction in cells grown on cellulose. The suppression by HGT-1/-2 was connected to both carbon catabolite repression (CCR) and the cyclic adenosine monophosphate-protein kinase A pathway. Alteration of a residue conserved across taxa in hexose transporters resulted in a loss of glucose-transporting function, whereas CCR signal transduction was retained, indicating dual functions for HGT-1/-2 as “transceptors.”

**Conclusions:**

In this study, GLT-1 and HGT-1/-2 were identified as the key components of the glucose dual-affinity transport system, which plays diverse roles in glucose transport and carbon metabolism. Given the wide conservation of the glucose dual-affinity transport system across fungal species, the identification of its components and their pleiotropic roles in this study shed important new light on the molecular basis of nutrient transport, signaling, and plant cell wall degradation in fungi.

**Electronic supplementary material:**

The online version of this article (doi:10.1186/s13068-017-0705-4) contains supplementary material, which is available to authorized users.

## Background

Organisms dynamically interact with their biotic and abiotic environments by adjusting cell transcriptional and post-transcriptional programs in response to external nutrient (like sugar) changes and stress stimuli. In theory, nutrient transport, sensing, and downstream signal transduction are critical for the coordination of extracellular nutrient availability with internal metabolism, development, and cell survival [1–3]. Glucose acts as a primary carbon source as well as a pivotal signal that triggers a cellular regulatory network influencing sugar transporter expression, carbon catabolism, and biomass accumulation. In particular, glucose sensing and uptake are critical for cellulase expression regulation via carbon catabolite repression (CCR) [4, 5]; therefore, elucidating the molecular basis of glucose transport is critical for plant biomass deconstruction and bio-based chemical and fuel production. The glucose transport system of *Saccharomyces cerevisiae* is the best documented system among fungal species, and consists of 20 different hexose carriers belonging to the major facilitator superfamily (MFS) [3, 6, 7]. *S. cerevisiae* possesses a dual-affinity transport system for glucose uptake that is coordinated with these hexose transporters composed of a high-affinity system (*K*_m_ = 1–2 mM) and a low-affinity system (*K*_m_ = 15–20 mM) [7]. In the filamentous fungi *Neurospora crassa*, glucose uptake also behaves in a dual-affinity fashion, with *K*_m_ values of 10–50 µM (system II) and 8–20 mM (system I) [8–10]. However, the genes of this dual system have still not been uncovered and their functions thus remain to be dissected.

Among the 20 different hexose carries in *S. cerevisiae*, two transporter-like glucose receptors Rgt2p and Snf3p, which sense low and high levels of external glucose, respectively, mediate a glucose signaling pathway [11, 12]. The recently identified high-affinity hexose transporter Hxt1, the homolog of Rgt2p/Snf3p in *Ustilago maydis*, also functions in glucose signaling [13]; this indicates the potential of hexose transporters to act as “transceptors” in fungi. Transceptors of other nutrients, such as nitrate, amino acids, phosphate, and cellobiose, have been found in eukaryotes [14–17], but glucose transceptors, to our knowledge, have not yet been definitively characterized, although Hxt1 in *Ustilago maydis* and GLUT2 in humans might possess this dual function [13, 18].

Another branch of glucose-sensing and signal modulation in fungi is mediated by the cAMP-protein kinase A (PKA) signaling pathway. In *S. cerevisiae*, binding of glucose to Gpr1p activates the downstream Gα protein Gpa2p, leading to stimulation of the adenylate cyclase Cyr1p, which increases cAMP levels, thereby affecting PKA activity [19]. The PKA pathway regulates a wide range of processes in fungi, including metabolism, cell growth, circadian rhythms, germination, and conidiation (for details, see reviews [19, 20]).

In glucose signaling in *N. crassa*, the ortholog of yeast Gpr1p is GPR-4 (NCU06312), which has been identified as a carbon source receptor [21]. Ligand binding to GPR-4 can stimulate the downstream Gα protein GNA-1 (NCU06493), leading to an increase in the level of cAMP produced by the activated adenylate cyclase CR-1 (NCU08377) [21]. External glucose sensing is associated with a Rgt2p/Snf3p ortholog RCO-3 (NCU02582)-mediated pathway, in which RCO-3 appears to act as a non-transporting glucose sensor [22]. Mutation of *rco*-*3* leads to complete dysfunction of the low-affinity transport system and partial impairment of the high-affinity system [22].

In this study, we characterized the glucose dual-affinity transport system in the model fungus *N. crassa*. One low-affinity glucose transporter, GLT-1 (NCU01633), and two high-affinity transporters, HGT-1/-2 (NCU10021 and NCU04963), were identified as the major components of systems I and II, respectively. Simultaneous deletion of *hgt*-*1/*-*2* (strain Δ*2hgt*) resulted in a notable increase in cellulolytic enzyme production when *N. crassa* was grown on cellulose. A group of carbohydrate-active enzymes (CAZys), nearly all glycolytic enzymes and asexual sporulation genes, were differentially expressed in the Δ*2hgt* mutant according to RNA-Seq. Based on analyses of an array of mutants and point mutations within HGT-1/-2, we hypothesized that the glucose dual-affinity transport system comprising GLT-1 and HGT-1/-2 in *N. crassa* was involved in glucose transportation, sensing, and downstream signaling cascades. This is the first time the glucose dual-affinity transport system of *N. crassa* has been systematically elucidated at the gene level. Our findings significantly improve our understanding of glucose uptake and signaling in fungi, and shed new light on plant cell wall deconstruction for cellulosic biorefinery by filamentous fungi.

## Results

### Genome-wide analysis of the transcriptional responses of fungal cells to a glucose gradient

**Fig. 1:**
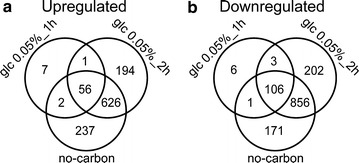
Comparative analysis of differentially expressed genes under glucose-depleted and glucose-free conditions vs. adequate glucose conditions (2.0%). *Venn diagram* analysis of (**a**) upregulated and (**b**) downregulated genes on 0.05% glucose after 1 and 2 h, and under no-carbon conditions

Transmembrane proteins are tightly regulated to coordinate environmental nutrient changes with intracellular metabolism and cell proliferation. Previous studies have suggested that the predicted low-affinity glucose transport system (system I) in *N. crassa* [8, 23, 24] is induced at high glucose levels, while glucose-limited or carbon-deprived conditions trigger de novo protein synthesis of the predicted high-affinity system (system II), which is fully activated within 1–2 h [9, 23]. These findings indicate that de novo mRNA synthesis of system II components occurs when mycelia are exposed to carbon-limited conditions. To obtain a broad view of the mode of expression and to define the functional molecular elements of the dual-affinity transport system, we conducted high-throughput sequencing (RNA-Seq) of wild-type (FGSC2489) mycelia exposed to a gradient of glucose (0, 0.05, 0.5, 2.0, 10.0%) for 1 or 2 h. To our knowledge, this is the first time transcriptional profiling analysis of a fungal response to a glucose gradient has been conducted. Sample-to-sample clustering [25] demonstrated the biological replicates were reliable, as evidenced by a high Spearman’s rho (>0.98, *P* value < 0.001) for all tested samples (Additional file 1: Figure S1a). Furthermore, the response observed under no-carbon conditions in this study was in good accordance with the published data [26] (Spearman’s rho = 0.96, *P* value < 0.001; Additional file 1: Figure S1B). Differential gene expression analysis (|GFOLD| > 1 and *P* value < 1 × 10^−4^, “Methods” section; genome-wide expression levels and differential expression analysis are described in Additional file 2: Table S1) revealed that the transcriptomic responses to 0.5% (27.8 mM), 2.0%, and 10.0% glucose were essentially similar (Table 1), indicating that a glucose threshold of 0.5% is adequate to support vegetative growth of *N. crassa* (note: all cited glucose percentages are *w/v* unless otherwise indicated). Although 96 genes with altered expression levels were detected on 10.0% glucose compared with 2.0%, no specific functional categories relevant to this mild osmotic stress condition (with reference to typical osmotic stress treatments of 3.0–8.0% NaCl in *S. cerevisiae* [27]) were enriched according to FunCat analysis [28] (Additional file 3: Table S2). In contrast, several categories associated with 66 genes robustly induced by 0.05% glucose for 1 h (compared with 2.0% glucose) were overrepresented (Additional file 3: Table S2). Among these categories were several reflecting cellular efforts to maintain homeostasis, including carbohydrate and fatty acid metabolism, glycolysis/gluconeogenesis, pentose utilization, sensing of external changes, and especially nutrient and ion transport. In the last category, 15 genes were annotated as transmembrane transporters based on TransportDB [29] (Additional file 3: Table S2). Additionally, 66 genes were predominant among the genes commonly upregulated in both 0.05% glucose for 2 h and carbon-free conditions (Fig. 1a; Additional file 3: Table S2), suggesting they belong to pioneer blocks in the response of the cellular architecture to external glucose depletion. This inference was especially supported by the strikingly decreased expression of the CCR regulator *cre*-*1* and its corepressor *rcm*-*1* (Additional file 2: Table S1), whereas the ortholog of Snf1, *prk*-*10* (NCU04566), was robustly upregulated under severe glucose-depleted conditions (Additional file 2: Table S1). In *A. nidulans*, activated SnfA can phosphorylate CreA, leading to CreA dissociation from the CreA-Ssn6-RcoA complex and consequent attenuation of CCR [30]. In addition to the attenuation of CCR, the cAMP-PKA glucose induction pathway seemed to have been downregulated, as both parallel cAMP synthetic processes were transcriptionally repressed. In particular, *gna*-*1* (NCU06493) and two Ras proto-oncogenes, *ras*-*1/*-*2* (NCU08823 and NCU06111), were 3- to tenfold downregulated under glucose-deprived or carbon-free conditions (Additional file 2: Table S1). In addition to cAMP-PKA inactivation, the transcriptomic downregulation also included reduced expression of DNA, RNA, and ribosomal biosynthetic genes, indicating that the fungal cells were struggling with carbon starvation (Fig. 1b; Additional file 3: Table S2). Two key autophagy genes, NCU00188 and NCU01545, encoding orthologs of ATG1/AtgA and ATG8/AtgH in *S. cerevisiae* and *A. nidulans*, respectively [31], showed increased expression levels under the no-carbon condition in comparison with 2.0% glucose treatment (Additional file 2: Table S1), whereas *vib*-*1* (NCU03725), an ortholog of the crucial autolysis regulator XprG in *A. nidulans* [32], was dramatically downregulated (Additional file 2: Table S1). These data suggest complex coordination between autophagy and autolysis in *N. crassa* to cope with carbon starvation as these two processes might be activated independently [32].

**Table 1:** Differentially expressed genes in the presence of a glucose gradient vs. 2.0% glucose

	No carbon	Glc 0.05%_2h	Glc 0.05%_1 h	Glc 0.5%_1 h	Glc 10%_1 h
Upregulated	921	877	66	0	41
Downregulated	1134	1167	116	0	55

### Identification and characterization of the glucose dual-affinity transport system revealed it is coordinated with glucose homeostasis

**Fig. 2:**
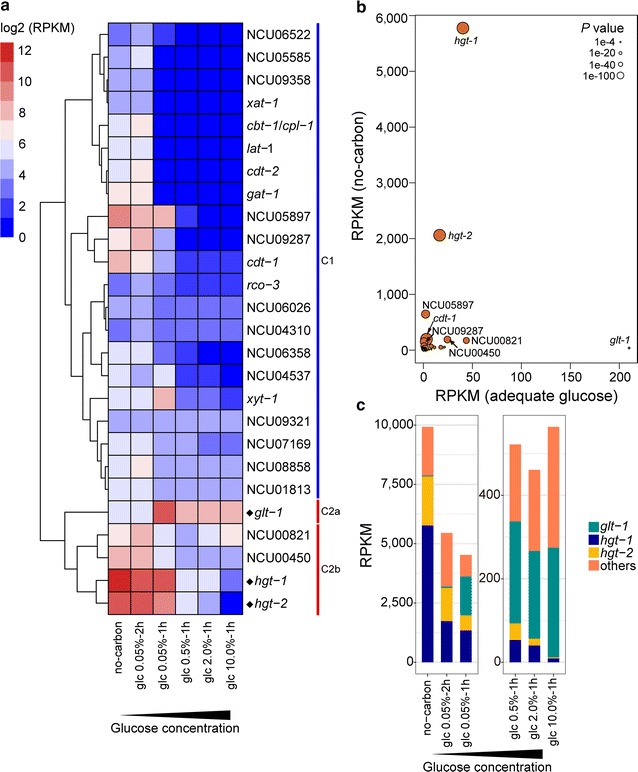
Transcriptional responses of sugar transporters to a glucose gradient in *Neurospora crassa*. **a** Heatmap analysis and clustering of 26 sugar transporters with robust expression levels (RPKM > 20) under at least one tested condition. Log-transformed expression values are color-coded. **b** Scatter plot of expression levels of all sugar transporters under no-carbon and adequate glucose (2.0%) conditions. Genes with RPKM values > 100 are indicated by their gene symbols. The area of the circle represents the *P* value of differential expression under no-carbon vs. 2.0% glucose conditions. **c** Expression comparison between potential candidates of the glucose dual-affinity transport system and all other putative sugar transporters

One of the most sensitive responses during external glucose depletion was related to nutrient assimilation (Additional file 3: Table S2). Previous work has shown that 39 putative sugar transporters are present in the genome of *N. crassa* [33, 34]. Among these transporters, 26 showed robust expression levels (i.e., reads per kilobase per million mapped reads [RPKM] > 20) under at least one tested treatment condition (Fig. 2a). Clustering analysis grouped eight identified MFS transporters into cluster 1 (C1), comprising one glucose sensor, *rco*-*3* [22]; two cellodextrin transporters, *cdt*-*1/*-*2* [35]; three pentose transporters, *lat*-*1* (also a weak glucose transporter), *xat*-*1*, and *xyt*-*1* [33, 36]; the galacturonic acid transporter *gat*-*1* [37]; and a cellobionic acid transporter, *cbt*-*1* [38]. C1 showed very little expression under 0.5% (or higher) glucose conditions; 17 out of 21 genes in this cluster displayed a weak response to external glucose changes, except *cdt*-*1*, *xyt*-*1*, NCU05897 (a putative fucose transporter), and NCU09287 (Fig. 2a). In contrast, C2 expression was more sensitive to glucose fluctuations. Included in C2 were the glucose transporter *glt*-*1* [33], the xylose-specific transporter NCU00821 [39], the putative sucrose transporter NCU00450, the high-affinity glucose transporter *hgt*-*1* [40], and NCU04963 (designated as high-affinity glucose transporter *hgt*-*2*; see the next paragraph). The glucose transporter *glt*-*1* was the only transporter that was constantly expressed on high glucose (Fig. 2), suggesting a predominant role in the low-affinity transport system. In contrast, *hgt*-*1/*-*2* were strongly upregulated under low-glucose or glucose-deprived conditions vs. 2.0% glucose. Furthermore, the mRNA expression levels of these two genes accounted for nearly 80% of the total expression of all sugar transporters under the no-carbon condition (Fig. 2b, c). This observed dynamic expression pattern demonstrated that *hgt*-*1/*-*2* were sensitively triggered by glucose depletion (Fig. 2c). Intriguingly, *glt*-*1* was predominantly expressed at high levels of glucose, but transiently strong upregulation was also observed under a moderately low-glucose concentration (0.05%, 1 h) (Fig. 2c), which suggests that its expression might be regulated by glucose in a dosage-controlled, bidirectional way.

**Fig. 3:**
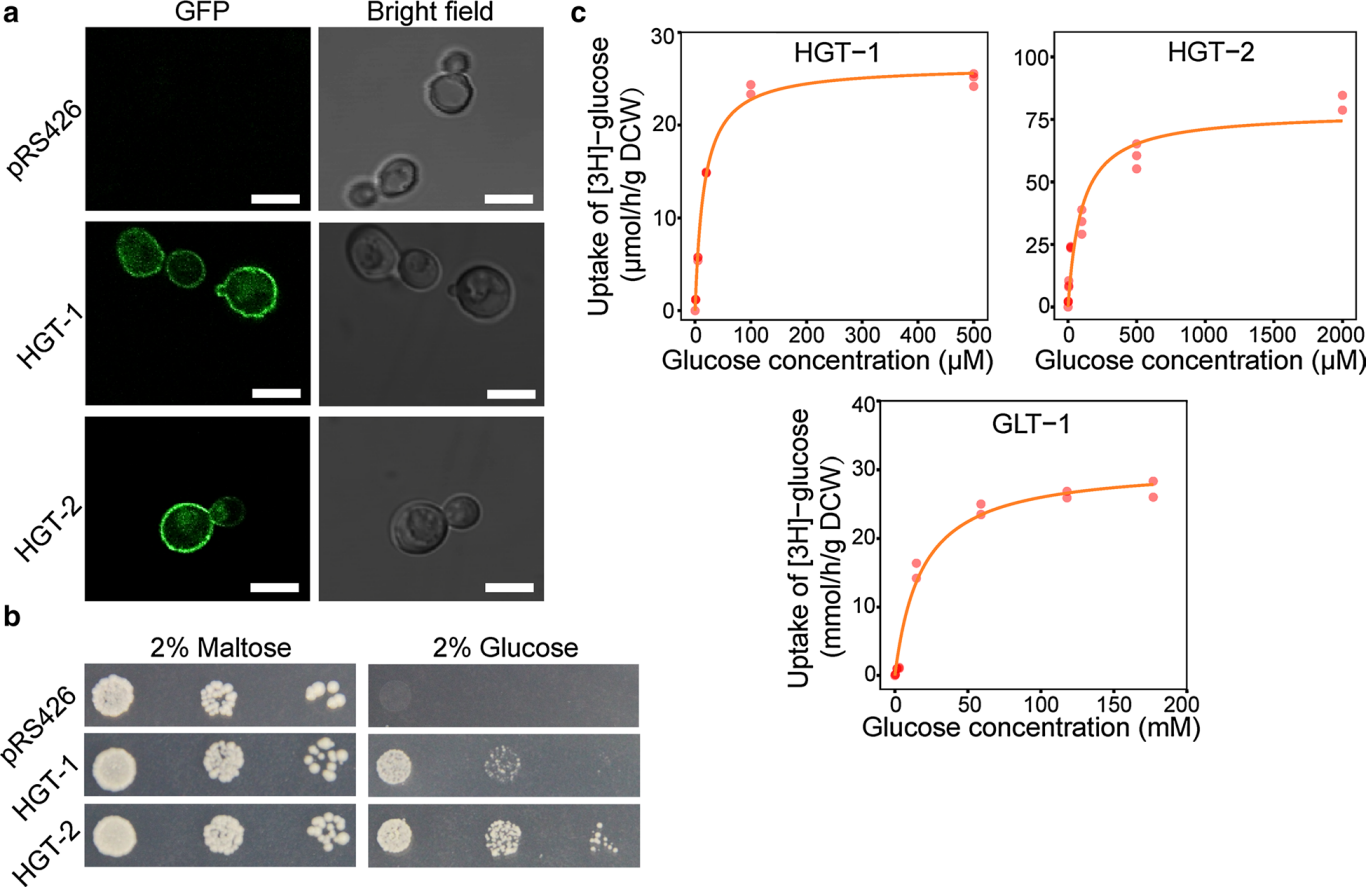
Subcellular localization and functional transport analysis of glucose transporters in *Saccharomyces cerevisiae*. **a** Glucose transporter coding sequences fused with the enhanced green fluorescence protein (eGFP) were expressed under the control of the *S. cerevisiae pgk1* promoter. A clone carrying the empty vector (pRS426) was the negative control. The *scale bar* corresponds to 5 µm. **b** Complementary growth of EBY.VW4000 harboring the indicated glucose membrane carriers on maltose or glucose for 3 or 6 days, respectively. **c** Michaelis–Menten kinetics of radiolabeled glucose uptake by *S. cerevisiae* containing HGT-1, HGT-2, or GLT-1

Because the *glt*-*1* mRNA was predominant on high glucose, while *hgt*-*1/*-*2* mRNAs dominated glucose-limited conditions (Fig. 2), these three transporters were selected as potential candidate genes for systems I and II, respectively. GLT-1 has been characterized as a glucose transporter that can efficiently complement the growth of the *S. cerevisiae* null-hexose-transporter strain EBY.VW4000 [41]. As anticipated, HGT-1/-2 functioned as glucose transporters when heterologously expressed in EBY.VW4000 (Fig. 3a, b). Uptake analysis with radiolabeled glucose in *S. cerevisiae* revealed a high *K*_m_ value for GLT-1 (18.42 ± 3.38 mM), while the *K*_m_ values for HGT-1 and HGT-2 were three orders of magnitude lower (16.13 ± 0.95 and 98.97 ± 22.02 µM, respectively) (Fig. 3c). This result was in line with a previous observation for HGT-1 [40]. The high *K*_m_ value observed for GLT-1 was comparable to the apparent affinity of system I (*K*_m_ = 8–20 mM), while the values of HGT-1/-2 were consistent with that of system II (10–50 µM). Summarizing the above data (Figs. 2, 3), GLT-1 was assigned to the low-affinity system (system I) and HGT-1/-2 to the high-affinity system (system II).

**Fig. 4:**
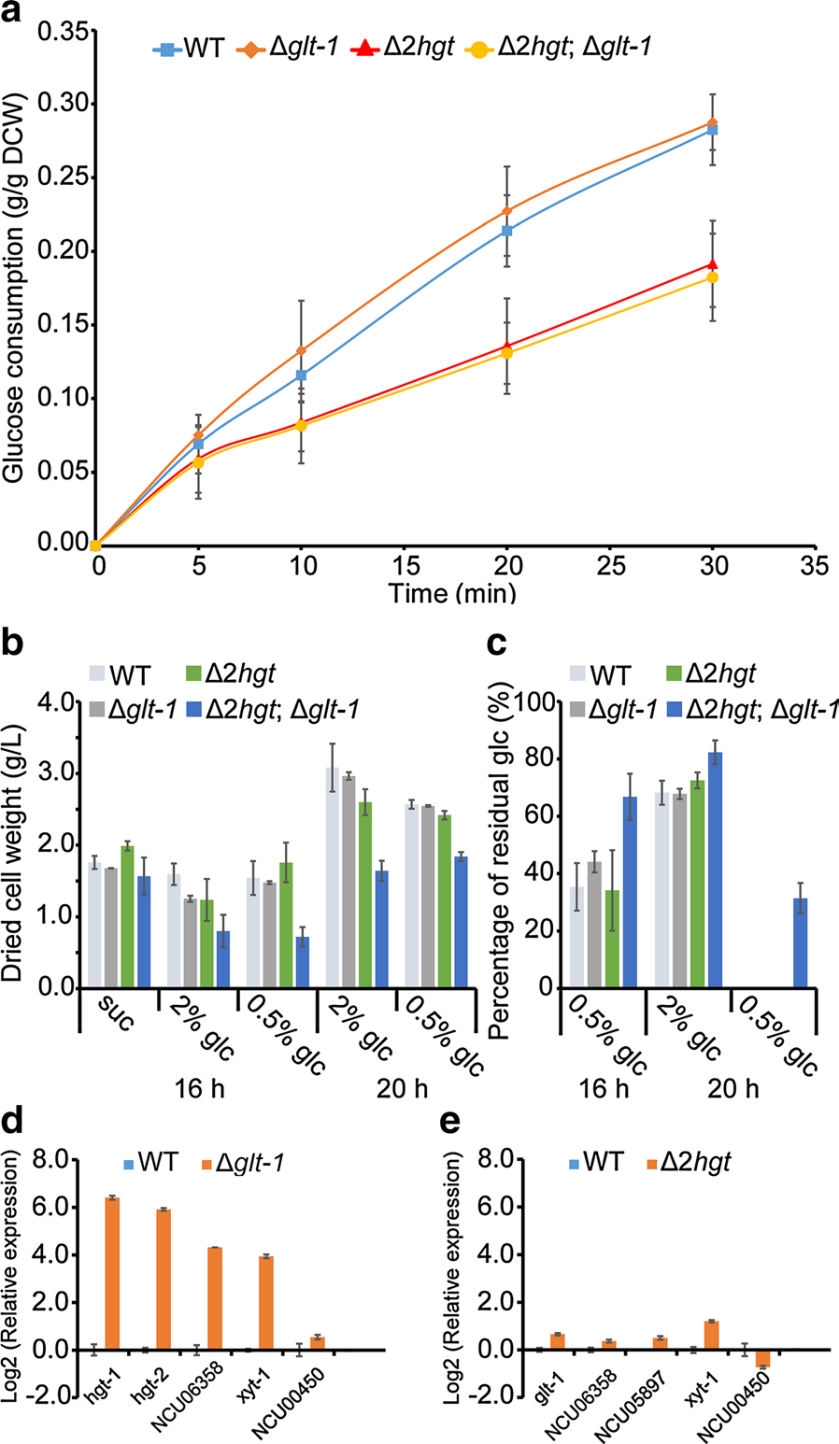
Phenotypes of Δ*glt*-*1*, Δ*2hgt*, and Δ*2hgt*;Δ*glt*-*1* mutants with respect to transient glucose uptake and vegetative growth on high levels of glucose. **a** Dynamic uptake curve of carbon-deprived mycelia of the key low- and/or high-affinity transporter-deleted strains under 1.1 mM glucose. Average values and standard deviations are shown for three biological replicates within at least two technical replicates for each sample. **b** Mycelial biomass and **c** residual glucose of the WT, Δ*glt*-*1*, Δ*2hgt*, and Δ*2hgt*;Δ*glt*-*1* after cultivation for the indicated time. **d** Relative expression levels of *hgt*-*1/*-*2* and several putative sugar transporters in the Δ*glt*-*1* mutant vs. the WT on 27.5 mM glucose. **e** Relative expression levels of *glt*-*1* and several putative sugar transporters in the Δ*2hgt* mutant *vs*. the WT on 0.55 mM glucose. Data are means of triplicate; error bars indicate standard deviations

To dissect the physiological functions of these major components of the glucose dual-affinity transport system, we generated double- and triple-gene deletion mutants via sexual crosses (three progenies were selected for the double mutant and two for the triple mutant in subsequent analysis; “Methods” section). Double deletion of *hgt*-*1/*-*2* resulting in the mutant Δ*hgt*-*1;*Δ*hgt*-*2* (designated Δ*2hgt* hereafter in the text) led to a significant deficiency in the uptake rate on a low concentration of glucose (1.1 mM; Fig. 4a). Further deletion of *glt*-*1* did not alter the transport capacity of the Δ*2hgt* mutant (Fig. 4a). This result was in agreement with the observation that the Δ*glt*-*1* strain had similar uptake activity to the WT (Fig. 4a). This similarity suggested that transport by GLT-1 (system I) was dispensable for glucose uptake under glucose-limited conditions, and that the high-affinity system (system II) was sufficient. When the putative sucrose transporter NCU00450 or NCU05897 (also strongly induced in low glucose; Fig. 2a) was also knocked out in the Δ*2hgt* background, no other significant difference was observed (Additional file 4: Figure S2). This result indicated that HGT-1/-2 were the major components of system II. In addition, the fact that the Δ*2hgt* mutant still exhibited detectable glucose uptake indicated that some other sugar transporters might have been responsible for limited glucose uptake when the two *hgt* genes were deleted (Fig. 4a). For example, LAT-1, which was slightly upregulated under no-carbon conditions (Fig. 2a), has been shown to have the ability to transport glucose in addition to its preferred substrate arabinose [36].

Intriguingly, deletion of *glt*-*1* did not lead to any deficiencies when *N. crassa* mycelia were grown on high levels of glucose (Fig. 4b, c), whereas the triple mutant Δ*2hgt*;Δ*glt*-*1* (Fig. 4b), in which all the major players of systems I and II were deleted, showed growth lag. This result indicated that system II could complement reduced activity of system I (Fig. 4c). To verify this conclusion, we performed a quantitative real-time reverse transcription polymerase chain reaction (qRT-PCR) assay to monitor gene expression in the Δ*glt*-*1* strain treated with high glucose. As expected, *hgt*-*1/*-*2* were dramatically upregulated (approximately 60-fold) in the presence of adequate glucose (0.5%; Fig. 4d). Some other putative sugar transporters, such as NCU06358 and *xyt*-*1* (Fig. 4d), were synchronously induced, indicating they have redundant roles in glucose uptake. In contrast, no significant increase in *glt*-*1* expression was found in the Δ*2hgt* strain at low levels of glucose (Fig. 4e).

### Negative influence of HGT-1/-2 on derepression of genes for plant cell wall deconstruction

Transcriptional expression data from previous reports [26, 33, 42, 43] and this study revealed a distinct utilization preference for glucose transporter systems in *N. crassa*: system I (*glt*-*1*) is activated when an adequate amount of the preferred carbon source is present, whereas system II (*hgt*-*1/*-*2*) is expressed under low-glucose, lignocellulosic, or carbon-free conditions (Additional file 5: Figure S3). Previous studies have demonstrated that system II can be derepressed by carbon-limited conditions [9, 10, 23], which was verified by our transcriptome profiling data (Fig. 2). Considering that a synergistic carbon starvation response can be induced by Avicel or plant biomass [26, 43], the high expression levels of *hgt*-*1/*-*2* observed in this study likely resulted from the lifting of CCR when mycelia were exposed to plant cell walls.

**Fig. 5:**
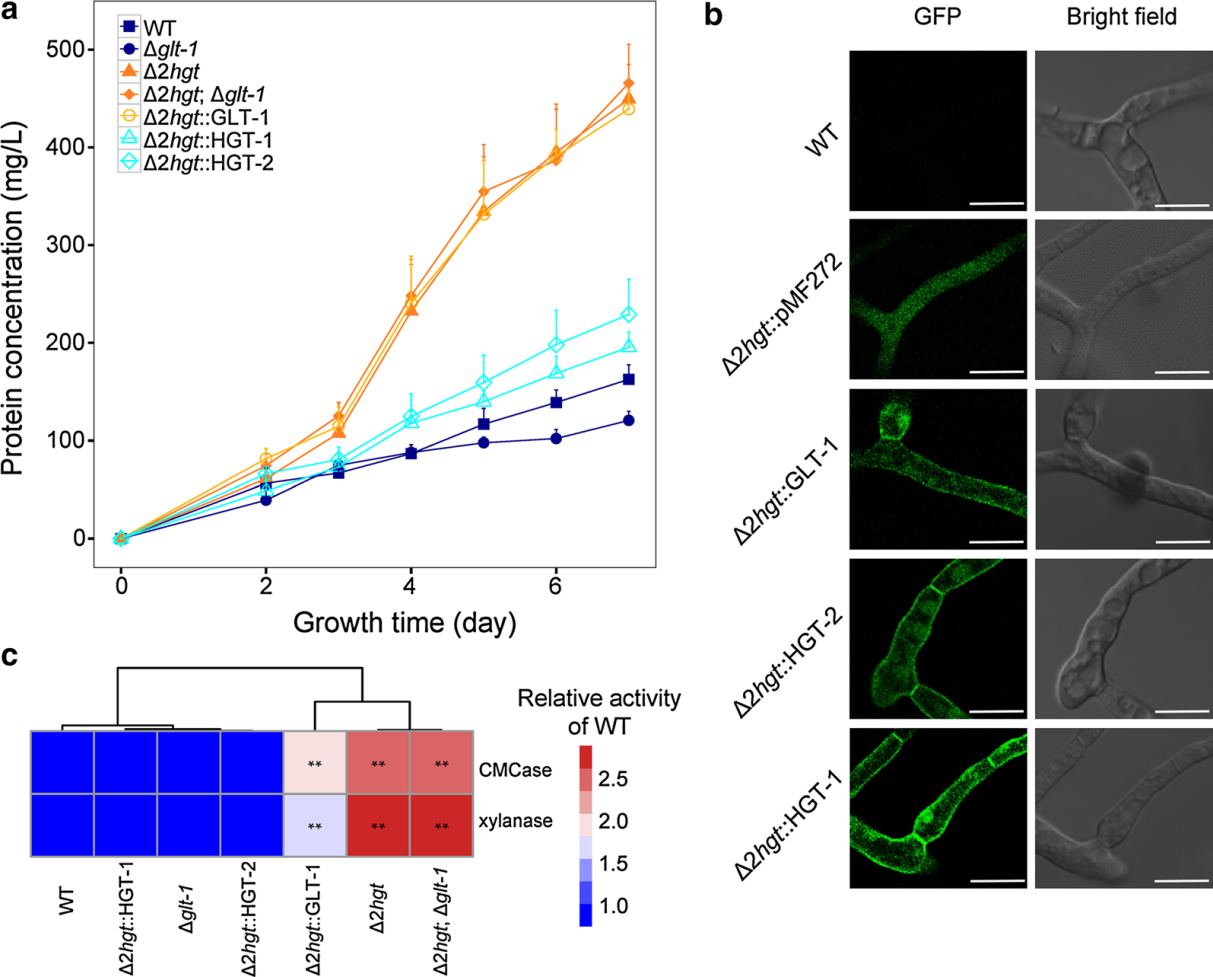
Distinct roles of GLT-1 and HGT-1/-2 in plant cell wall-degrading enzyme production. **a** Secreted protein production of Avicel cultures of the WT, Δ*glt*-*1*, Δ*2hgt*, Δ*2hgt*;Δ*glt*-*1*, and a series of Δ*2hgt*::glucose transporter strains. The means and standard deviations of triplicate are shown. **b** Subcellular localization of HGT-1, HGT-2, and GLT-1 in *Neurospora crassa*. A transformant harboring pMF272-eGFP was the negative control. The *scale bar* corresponds to 10 µm. **c** Cellulase activity (CMCase) and xylanase activity per gram dried cell weight of Avicel culture supernatants at 7 days. Relative activities vs. the WT are color-coded. The statistical significance of mutant values compared with the WT was determined using a two-tailed Student’s *t*-test; *P < 0.05, **P < 0.01

Given that *hgt*-*1/*-*2* are strongly derepressed under cellulosic conditions, we wondered whether these two genes play roles in cellulose utilization in *N. crassa*. To answer this question, we inoculated a series of *hgt*-deleted mutants and the WT into liquid medium with 2.0% (*w/v*) Avicel as the sole carbon source. Surprisingly, the Δ*2hgt* strain showed nearly twofold higher protein production than the WT (Fig. 5a), while only slightly improved secretion rates were found in the single *hgt*-deleted mutants (Additional file 6: Figure S4). The triple mutant Δ*2hgt*;Δ*glt*-*1* displayed hyper-production similar to that of Δ*2hgt*, consistent with the glucose uptake observed in Fig. 4a, with similar biomass accumulation to the WT (Additional file 7: Figure S5). To verify that the two high-affinity glucose transporters (HGT-1/-2) but not the low-affinity one (GLT-1) affected lignocellulase expression, the three transporters driven by the promoter of *ccg*-*1*, which is highly expressed under cellulose- or carbon starvation conditions [26, 44] (Additional file 2: Table S1), were individually introduced into the Δ*2hgt* mutant (Fig. 5b). Both high-affinity transporters, HGT-1/-2, restored the lignocellulase production and activity of Δ*2hgt* to WT levels, but the low-affinity transporter GLT-1 did not (Fig. 5a, c; Additional file 8: Table S3). These data demonstrate that the derepressible high-affinity glucose transport system negatively affects cellulase expression in *N. crassa*.

**Fig. 6:**
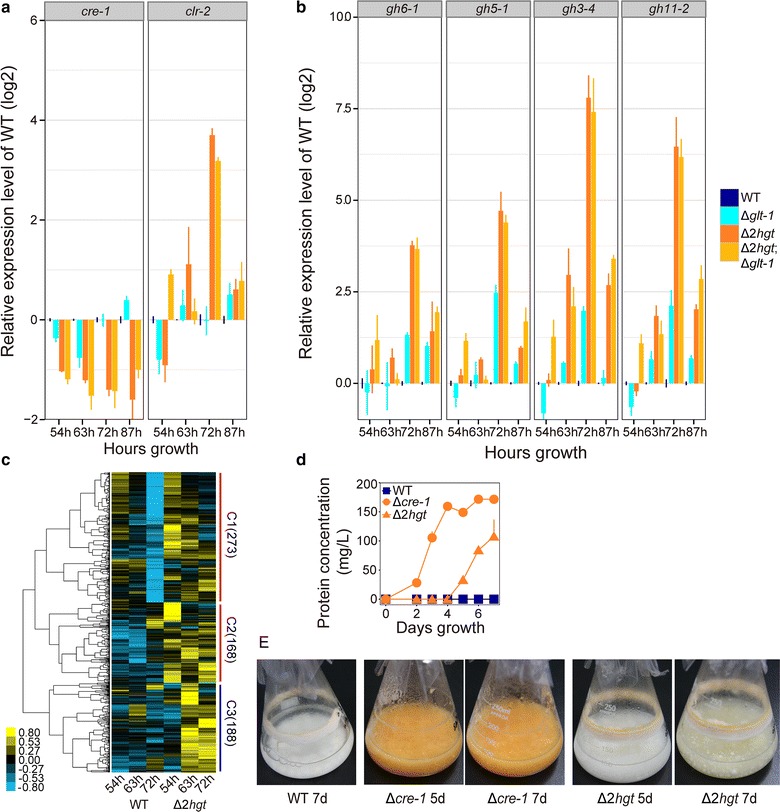
Simultaneous deletion of HGT-1/-2 leads to derepression of genes encoding lignocellulases and relief of catabolite repression. Relative expression levels of **a** two transcription factors (*cre*-*1* and *clr*-*2*) and **b** lignocellulases (*gh6*-*1*, *gh5*-*1*, *gh3*-*4*, and *gh11*-*2*) in Δ*glt*-*1*, Δ*2hgt*, and Δ*2hgt*;Δ*glt*-*1* mutants vs. the WT after 2–4 days growth on Avicel. Average values and standard deviations of two biological replicates/two technical replicates are shown. **c** Hierarchical clustering of RPKM values for 629 differentially expressed genes in Δ*2hgt* vs. the WT. The gene counts of each group are shown in brackets. **d** Secreted protein production of Avicel plus 0.1% 2-DG cultures of the WT, Δ*2hgt*, and Δ*cre*-*1*. The means and standard deviations of triplicate are shown. **e** Growth phenotypes of the cultures in *panel* D

Transcriptional analysis by qRT-PCR revealed that expression levels of the cellulase regulator *clr*-*2* (NCU08042) and the tested carbohydrate-active enzymes (CAZy genes) were considerably higher in Δ*2hgt* and Δ*2hgt*;Δ*glt*-*1* compared with the WT (Fig. 6a, b). The largest differences from the WT (Fig. 6b) were observed 3 days after conidial inoculation, at around the time protein production started to deviate upwards in the Δ*2hgt* mutant (Fig. 5a).

To further elucidate the genome-wide expression changes in Δ*2hgt*, time-course comparative transcriptome profiling was performed via RNA-Seq. Correlation analysis showed reliable biological reproducibility as indicated by sample-to-sample clustering and a high Spearman’s rho (>0.96, *P* value < 0.001) (Additional file 9: Figure S6; gene expression levels and differential expression analysis are shown in Additional file 10: Table S4). Overall, 629 genes were robustly upregulated in the system II-deficient mutant compared with the WT at the three tested time points (Fig. 6c; Table 2). Hierarchical clustering analysis of the 629 genes revealed three clusters (Fig. 6c). The first cluster (C1) contained 237 genes whose expression levels decreased in both the WT and the Δ*2hgt* mutant with increasing culture time, but were generally higher in Δ*2hgt* compared with the WT at each tested time point (Fig. 6c). Most upregulated CAZy genes from the 629 gene set were overrepresented in C1 (57 out of 70, *P* = 6.65 × 10^−5^ according to the one-tailed Fisher’s exact test; Additional file 11: Table S5). Also included in this group was *clr*-*2* (Additional file 11: Table S5). Although *clr*-*1* did not meet the strict differential analysis criteria (“Methods” section) with respect to the *hgt* null mutant (GFOLD = 0.43), it displayed a significantly elevated expression level at 72 h (*P* = 6.88 × 10^−19^ and raw log_2_[fold change] = 1.42; Additional file 10: Table S4). In *N. crassa*, the regulators *clr*-*1/*-*2* positively control the transcription of a number of lignocellulases, among which 126 genes are defined as belonging to the “Avicel regulon” [26]. Fifty-seven such genes were found in the 629 gene set, 53 of which were enriched in C1. This finding suggests that the upregulated genes in C1 are at least partially regulated by *clr*-*1/*-*2*. Functional category enrichment analysis also supported this idea (Additional file 11: Table S5). The group C1 also contained 11 MFS transporters, including *glt*-*1*. This sugar transporter and another one in C2 (*xyt*-*1*) were synergistically transcribed to high levels in the Δ*2hgt* mutant (Additional file 10: Table S4).

**Table 2:** Differentially expressed genes in the Δ*2hgt* mutant grown on cellulose compared with the wild type

Hours growth	40 h		54 h		72 h		Sum	
	Total	CAZy gene	Total	CAZy gene	Total	CAZy gene	Total	CAZy gene
Upregulated	128	12	305	18	378	60	629	70
Downregulated	135	1	78	6	265	8	384	15

In addition to many derepressed lignocellulases, the pentose phosphate pathway was overrepresented in C1 (*P* = 4.03 × 10^−4^; Additional file 11: Table S5). This observation supports the notion that CCR was lifted when *hgt*-*1/*-*2* were deleted. In fact, the expression level of *inv* (NCU04265), an invertase that has been widely demonstrated to be a marker for CCR in *N. crassa* and yeast species [45–47], was consistently high in Δ*2hgt* (Additional file 10: Table S4). Furthermore, *cre*-*1*, a well-characterized regulator involved in CCR that globally suppresses cellulase expression in *N. crassa* [4], was downregulated in Δ*2hgt* (Fig. 6a). To confirm that CCR was lifted in the Δ*2hgt* mutant, the glucose analog 2-deoxy-glucose (2-DG, which cannot be catalyzed during glycolysis and is a drug often used for glucose repression analysis in filamentous fungi) was added to the Vogel’s growth medium containing Avicel as the sole carbon source. Supporting the above hypothesis, the Δ*2hgt* strain showed less sensitivity to 2-DG relative to the WT (Fig. 6d, e). Taken together, these results indicate that HGT-1/-2 contribute to the activation of CCR in the WT at a certain time point during the cellulolytic utilization process, including activation of *cre*-*1* expression, which triggers general repression affecting *clr*-*1/*-*2* and thus suppressing lignocellulase gene expression.

### Lignocellulase gene repression by HGT-1/-2: functionally coordinated but not confined to CRE-1

**Fig. 7:**
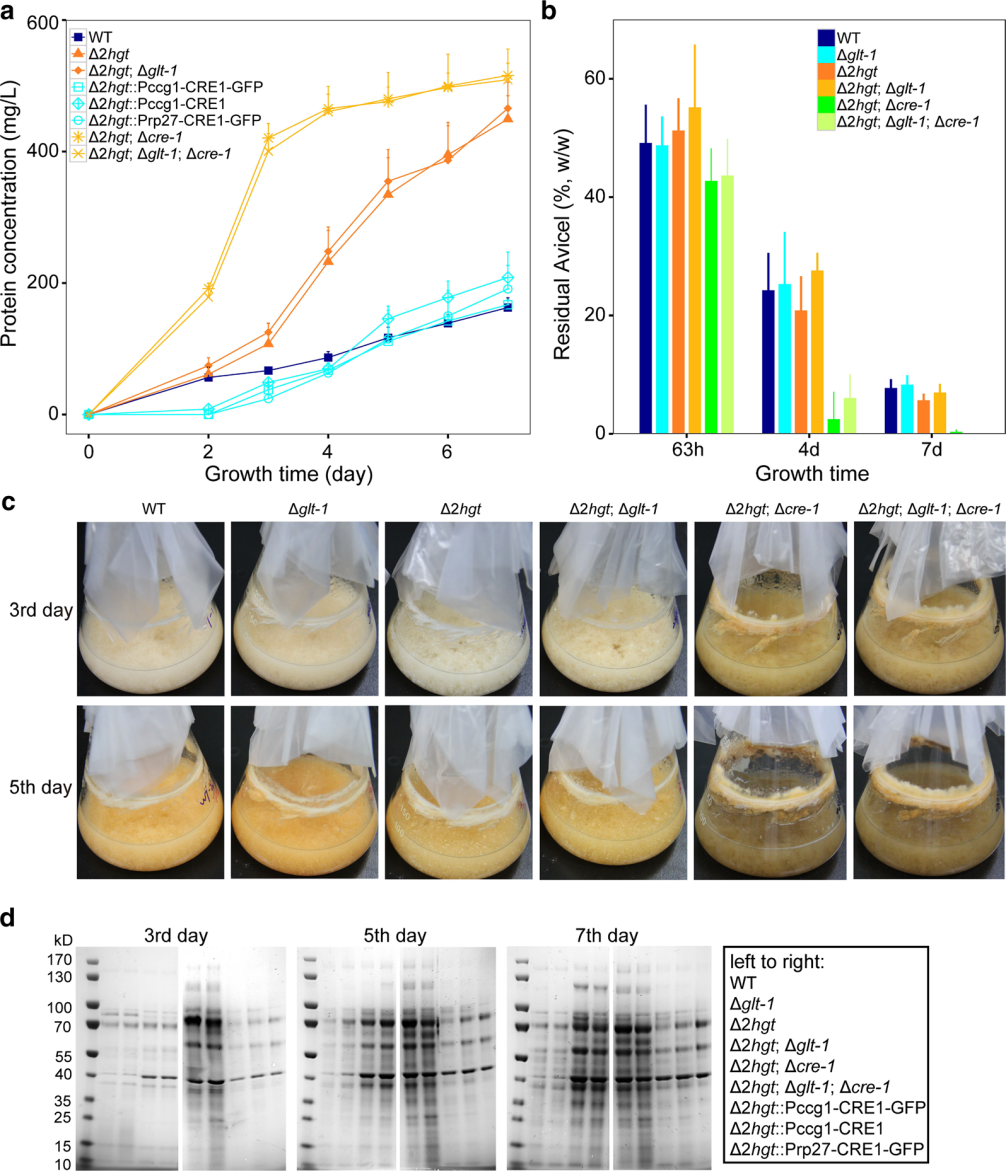
The CCR regulator CRE-1 is involved in the repression modulated by HGT-1/-2. **a** Protein concentrations of Avicel cultures of the WT, Δ*2hgt*, Δ*2hgt*;Δ*glt*-*1*, Δ*2hgt*;Δ*cre*-*1*, Δ*2hgt*;Δ*glt*-*1*;Δ*cre*-*1*, and a series of Δ*2hgt*::CRE-1 strains. **b** Residual Avicel in cultures of WT, Δ*glt*-*1*, Δ*2hgt*, Δ*2hgt*;Δ*glt*-*1*, Δ*2hgt*;Δ*cre*-*1*, and Δ*2hgt*;Δ*glt*-*1*;Δ*cre*-*1* strains. **c** Growth phenotypes and **d** secretomes of the cultures in *panel* A. The means and standard deviations of triplicate are shown

As shown above, exposure to carbon-limited environments such as plant cell walls causes *N. crassa* to use the high-affinity transporter system to uptake cellulose-derived glucose. The observation that *cre*-*1* expression was reduced during cellulose utilization (Fig. 6a) therefore suggests that the suppression of lignocellulolytic degradation enzymes mediated by HGT-1/-2 might be downstream-regulated by CRE-1. To test this idea, *cre*-*1* was deleted or constantly expressed in the Δ*2hgt* mutant background. The rate of secreted protein production by the Δ*2hgt*;Δ*cre*-*1* and Δ*2hgt*;Δ*glt*-*1*;Δ*cre*-*1* mutants on Avicel was dramatically increased compared with the Δ*2hgt* strain (Fig. 7a). This difference was even more obvious when the Avicel substrate was replaced with cellobiose, on which deletion of *cre*-*1* resulted in protein production starting at least 3 days earlier and ultimately twofold greater accumulation than the background strain Δ*2hgt* (Additional file 12: Figure S7). Examination of submerged growth phenotypes and protein gels confirmed this observation (Fig. 7c, d). In accordance with these results, the consumption of Avicel by Δ*2hgt*;Δ*cre*-*1* was also faster than that of Δ*2hgt* and the WT (Fig. 7b). Previous work has demonstrated that the single *cre*-*1* deletion mutant utilizes the Avicel substrate 2–3 days faster than the WT [4], implying an independent role for *cre*-*1* in the Δ*2hgt* mutant during cellulose utilization. In the Δ*2hgt* strain (Additional file 13: Figure S8), however, constant activation of *cre*-*1* expression via the *ccg*-*1* promoter or the ribosomal protein 27 promoter from *Magnaporthe grisea* [48] suppressed extracellular protein production to a level comparable to that of the WT (Fig. 7a, d). This result implies that regulation of cellulase expression via CRE-1 is coordinated with HGT-modulated repression in *N. crassa*. All these data, combined with the observation of reduced *cre*-*1* expression in the Δ*2hgt* strain (Fig. 7a), suggest that repression by HGT-1/-2 involves cross-talk with the pivotal CCR regulator CRE-1 and potentially some other unknown factor(s).

**Fig. 8:**
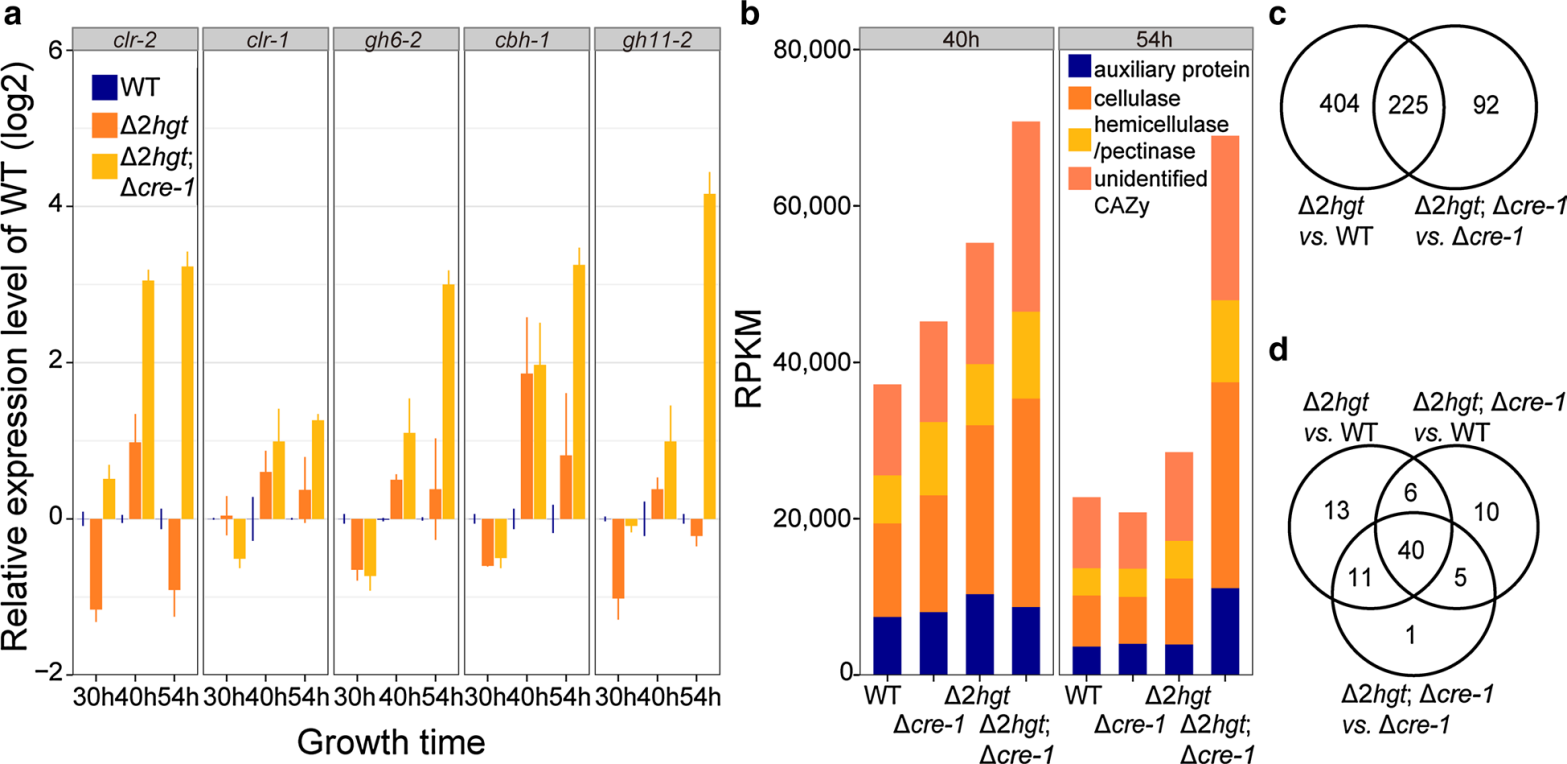
Comparative analysis of gene expression between Δ*2hgt* and Δ*2hgt*;Δ*cre*-*1* confirmed the existence of cross-talk between CRE-1 and HGT-modulated suppression. **a** Relative expression levels of two transcription factors (*clr*-*1* and *clr*-*2*) and lignocellulases (*gh6*-*2*, *cbh1*, and *gh11*-*2*) in Δ*2hgt* and Δ*2hgt*;Δ*cre*-*1* mutants vs. the WT after 30, 40, and 54 h growth on Avicel. Average values and standard deviations of two biological replicates/two technical replicates are shown. **b** Expression levels from RNA-Seq data of genes encoding different classes of CAZy proteins in WT, Δ*cre*-*1*, Δ*2hgt*, and Δ*2hgt*;Δ*cre*-*1* strains. **c** *Venn diagram* analysis of upregulated genes in Δ*2hgt vs.* the WT and Δ*2hgt*;Δ*cre*-*1* vs. Δ*cre*-*1*. **d** *Venn diagram* analysis of derepressed genes encoding CAZy proteins in Δ*2hgt* vs. the WT, Δ*2hgt*;Δ*cre*-*1* vs. the WT, and Δ*2hgt*;Δ*cre*-*1* vs. Δ*cre*-*1*

To verify our hypothesis, comparative genome-wide expression profiling was performed via RNA-Seq between the Δ*2hgt* and Δ*2hgt*;Δ*cre*-*1* mutants (Additional file 9: Figure S6; Additional file 10: Table S4). The expression levels of selected cellulases as well as total CAZome expression were greatly elevated in the Δ*2hgt*;Δ*cre*-*1* strain compared with the WT (Fig. 8a, b). The upregulated genes included those encoding typical “CRE-1 regulon” proteins—an α-amylase A (NCU09805), a starch-binding enzyme (NCU08746), *gh6*-*3* (NCU07190), the invertase *inv*, a glucoamylase (NCU01517), and a galactosidase (NCU00972) (Additional file 11: Table S5), which were all identified in *N. crassa* in a previous study [4] and implied the existence of CRE-1-mediated cross-talk in the Δ*2hgt* mutant as mentioned above. In contrast, 115 out of 128 upregulated genes in the Δ*2hgt* strain at the 40-h time point (Table 2) were not typical CRE-1 regulon members [4]. Furthermore, combined deletion of *hgt*-*1/*-*2* in the Δ*cre*-*1* background (strain Δ*2hgt*; Δ*cre*-*1*) resulted in upregulation of 317 genes, of which 225 overlapped with the 629 gene set identified in Δ*2hgt* (Fig. 8c; Table 3). CAZy proteins were also overrepresented in this overlapping gene set (51 out of a total of 70 in the 629 gene set) (Additional file 11: Table S5; Fig. 8d). This observation cannot be completely explained by the action of the CCR regulator CRE-1, and thus further suggests that one or more additional factors might be involved in modulating the HGT-1/-2 suppression signal that leads to the repression of cellulase expression.

**Table 3:** Comparative analysis of differentially expressed genes in the Δ*2hgt* and Δ*2hgt*;Δ*cre*-*1* mutants grown on cellulose

Hours growth	Comparison	Up in 629-genes (a)	Up in total (b)	Ratio (a/b) (%)	CAZy gene
40 h	Δ*2hgt*;Δ*cre-1* vs. WT	189	274	69.0	23
	Δ*2hgt*;Δ*cre-1* vs. Δ*cre-1*	84	120	70.0	15
54 h	Δ*2hgt*;Δ*cre-1* vs. WT	288	562	51.2	59
	Δ*2hgt*;Δ*cre-1* vs. Δ*cre-1*	182	240	75.8	54
Sum	Δ*2hgt*;Δ*cre-1* vs. WT	349	677	51.6	61
	Δ*2hgt*;Δ*cre-1* vs. Δ*cre-1*	225	317	71.0	57

### Significantly increased intracellular levels of cAMP in Δ*2hgt* under cellulosic conditions give rise to a negative regulation circuit in the cAMP-PKA pathway

**Fig. 9:**
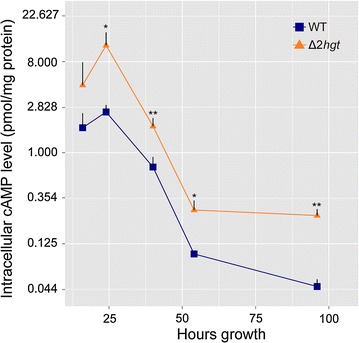
Intracellular cAMP is significantly increased in Δ*2hgt* under cellulosic conditions compared with the WT. Samples of the WT and the Δ*2hgt* mutant cultured for 16, 24, 40, 54, and 98 h were harvested and assayed for cAMP levels. Values represent the means of two independent biological replicates within each of three technical replicates; error bars show standard deviations. Statistical significance was assessed using a two-tailed Student *t* test. *P < 0.05, **P < 0.01

As shown above, one or more unknown factors seem to participate in the connection between HGT-modulated suppression and CAZy expression. The cAMP-PKA signaling pathway, which functions in the sensing of external or internal nutrient changes, is highly conserved across living kingdoms [18, 19]. In *N. crassa*, misactivation of PKA triggers a number of developmental events, such as apolar growth, aerial hyphal formation, vegetative growth, and improved protein production upon cellulose; in contrast, inactivation leads to premature conidiation, increased thermotolerance, and nearly abolished circadian rhythms [49–51]. Intriguingly, 11 genes involved in glycolysis, two in gluconeogenesis, and two fermentative genes were robustly derepressed in the Δ*2hgt* mutant, which implies that vegetative growth was facilitated under cellulosic conditions when *hgt*-*1/*-*2* were deleted (Additional file 11: Table S5). In contrast, a large number of sporulation-related genes were inactivated, including the critical regulator of minor chain formation *fl* (NCU08726) [52], the rodlet layer protein *eas* (NCU08457) [53], the conidiation-specific gene *con*-*10* (NCU07325) [54], the all development-altered regulator *ada*-*6* (NCU04866) [55], the *Aspergillus flbC* homolog NCU03184 [56], and the *Aspergillus flbD* ortholog *rca*-*1* (NCU01312) (Additional file 11: Table S5). The role of *rca*-*1* in conidiation is uncertain, but it was recently revealed to participate in lignocellulolytic enzyme production in *N. crassa* [43]. All these observations suggest that the cAMP-PKA pathway was activated by the double deletion of *hgt*-*1/*-*2*. To test this hypothesis, intracellular cAMP levels were measured in the WT and the Δ*2hgt* strain. As anticipated, cAMP levels during the cellulolytic utilization phase were significantly higher in the *hgt*-deleted mutant relative to the WT (Fig. 9). Additionally, *pkac*-*2* (NCU00682), the paralog of PKAC-1 in *N. crassa* [57], was upregulated in the *hgt*-deleted mutant compared with the WT at all three tested time points from 40 to 72 h (Additional file 11: Table S5), further supporting the involvement of the cAMP-PKA pathway in HGT-modulated suppression.

### Replacing a conserved arginine with lysine in HGT-1/-2 leads to dysfunction of glucose transport but no change in CCR signal transduction

**Fig. 10:**
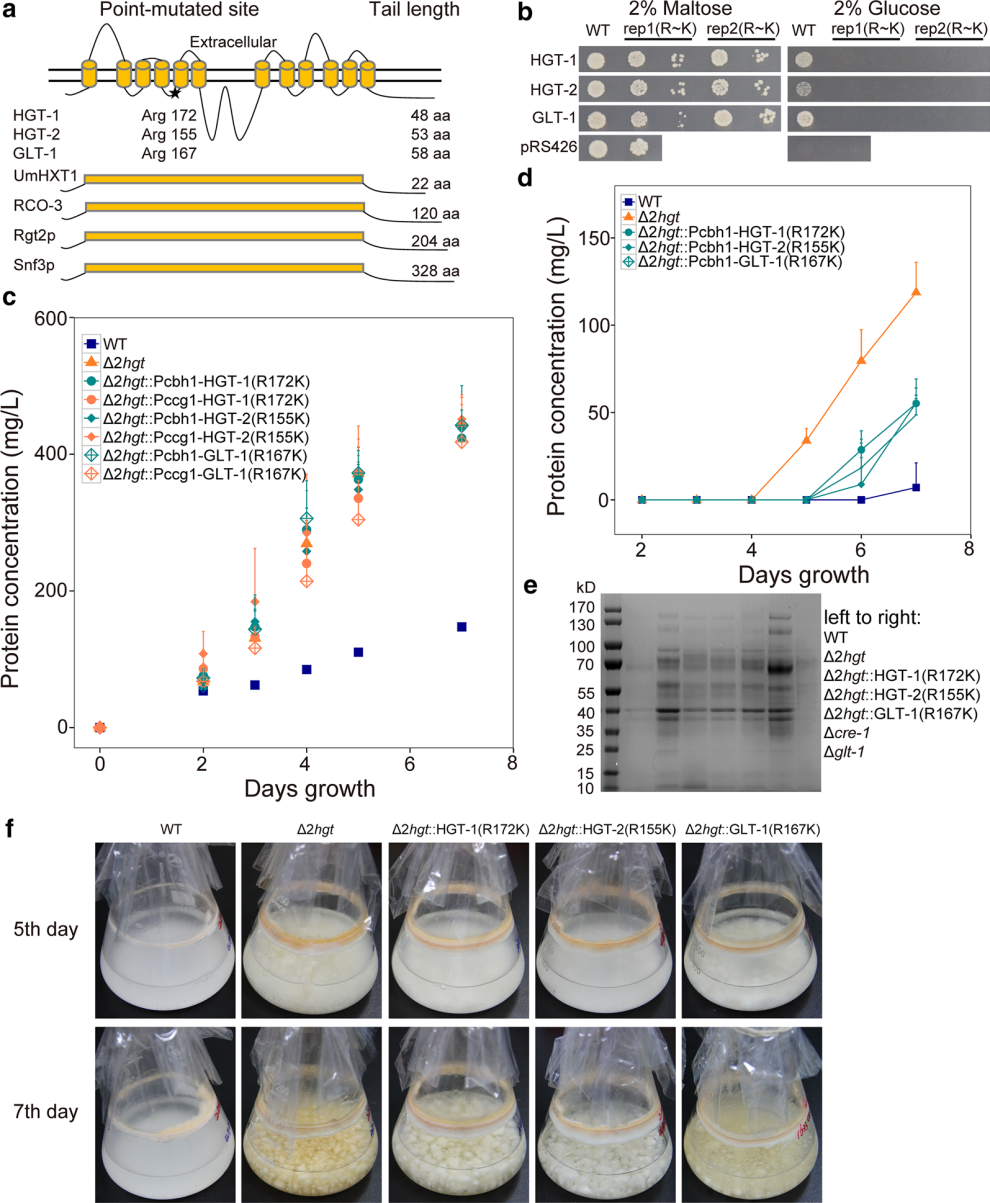
Evidence that non-transporting HGT-1/-2 analogs are functional in CCR signal transduction in Δ*2* *hgt*. **a** Structural models of HGT-1/-2 and GLT-1 based on TMHMM. One arginine that was replaced by lysine is marked with an *asterisk*. **b** Growth of EBY.VW4000 harboring the indicated wild-type transporters and corresponding point-mutated analogs on maltose or glucose as the sole carbon source. Each transporter analog was tested using two biological replicates. **c** Protein concentrations of Avicel cultures of the Δ*2hgt* mutant expressing one indicated analog controlled by the *ccg*-*1* or *cbh*-*1* promoter. Average values and standard deviations of triplicate are shown. **d** Protein concentrations, **e** secretomes, and **f** growth phenotypes on Avicel plus 2-DG of the mutant cultures in *panel* C

Various studies have found that replacing a conserved arginine with a lysine residue in yeast species and the fungal pathogen *U. maydis* leads to constitutive glucose signaling by hexose sensors, including Snf3p/Rgt2p, Hgt4, Hxs1, and UmHxt1 [11, 13, 58, 59]. To test whether HGT-1/-2 also showed a similar conserved function when this residue was altered, we generated the constructs HGT-1(R172K), HGT-2(R155K), and GLT-1(R167K) (Fig. 10a). Consistent with previous studies [13], these analogs no longer supported the growth of EBY.VW4000 when glucose was the sole carbon source (Fig. 10b). When the mutant proteins were separately introduced into the Δ*2hgt* strain, the extracellular protein levels were similar to the background strain on Avicel, seemingly implying complete loss of function via this point mutation (Fig. 10c). With the addition of 2-DG, however, the strains harboring the analogs of the two high-affinity glucose transporters (HGT-1/-2) were much more sensitive to 2-DG than the background strain (Δ*2hgt*) and the strain with mutated GLT-1. The latter two mutants, Δ*2hgt* and Δ*2hgt*::GLT-1(R167K), grew much better than the WT, Δ*2hgt*::HGT-1(R172K), and Δ*2hgt*::HGT-2(R155K) (Fig. 10f), indicating that the CCR signal was transmitted by the HGT-1/-2 analogs in the Δ*2hgt*::HGT-1(R172K) and Δ*2hgt*::HGT-2(R155K) strains. The strength of CCR in the mutants harboring HGT-1/-2 analogs was reduced compared with the WT (Fig. 10d, e), which may have been because a single *hgt* sequence could not fully complement the defect of the double deletion strain Δ*2hgt* (Fig. 5a). Taken together, these data supported the hypothesis that HGT-1/-2 possess an additional function as “glucose transceptors” in *N. crassa*.

### Conservation of the dual-affinity transport components from saprophytes to parasitic fungi

**Fig. 11:**
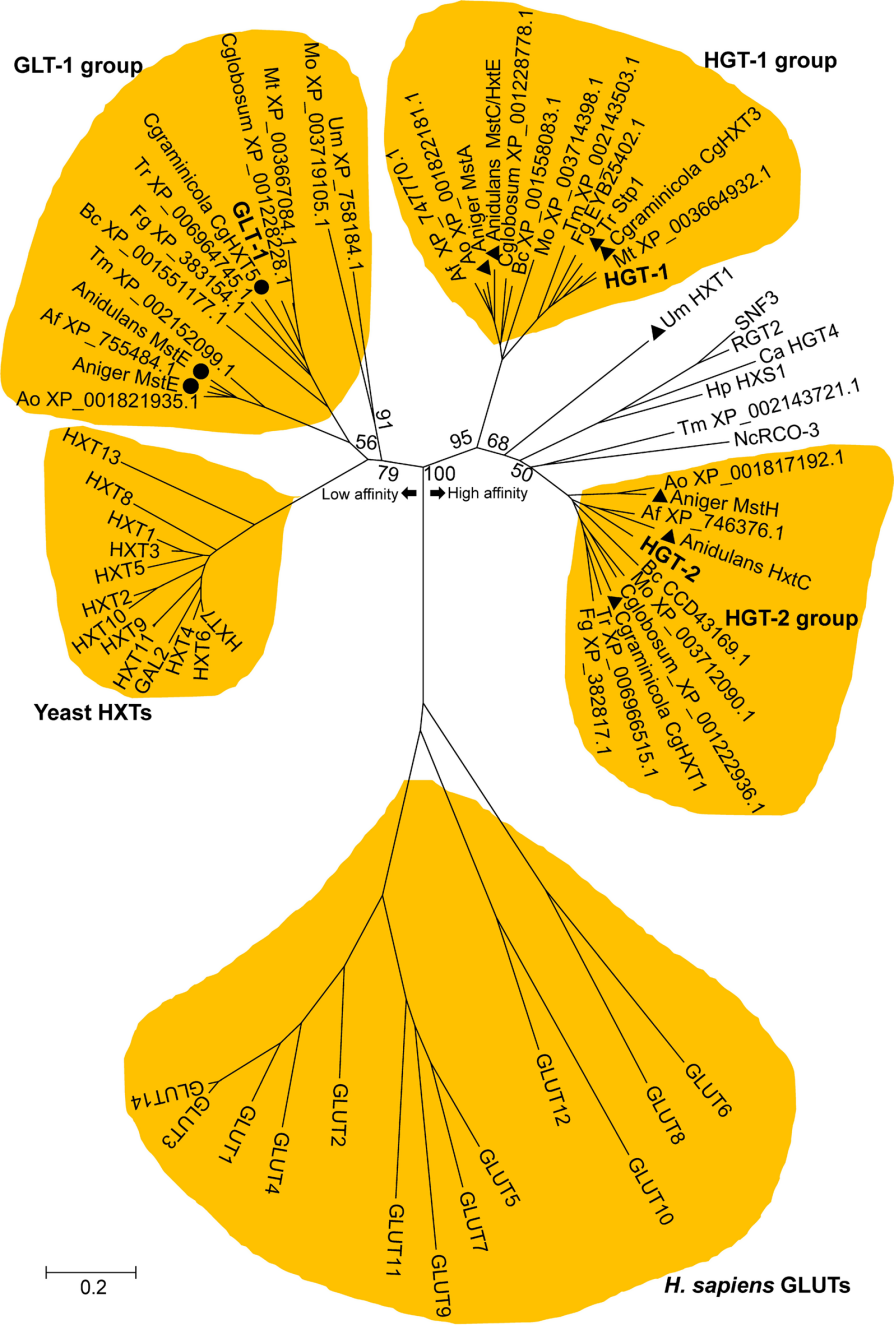
Phylogenetic analysis of GLT-1 and HGT-1/-2 homologs in the fungal kingdom. Characterized glucose transporters of the selected fungi are marked with *solid circles* (for low-affinity transporters) and *solid triangles* (for high-affinity transporters). Gene names or protein accession numbers are given. The glucose sensors Snf3p/Rgt2p, HGT4, and HXS1 from yeast, RCO-3 from *N. crassa*, and GLUTs from *H. sapiens* were included as outgroups; bootstrap support values based on 1000 replicates are shown at the major branches

To investigate the conservation of the glucose transport dual-affinity system in fungi, homologs of systems I and II proteins were identified using GLT-1 and HGT-1/-2 protein sequences as queries in searches against the genomes of saprophytes (*A. nidulans*, *A. niger*, *A. oryzae*, *Myceliophthora thermophila*, and *T. reesei*), phytopathogens (*Botrytis cinerea*, *Chaetomium globosum*, *Colletotrichum graminicola*, *Fusarium graminearum*, *Magnaporthe oryzae*, and *U. maydis*), and animal fungal pathogens (*A. fumigatus* and *Talaromyces marneffei*) (“Methods” section). A phylogenetic analysis was conducted that included hexose transporters from *S. cerevisiae* (Fig. 11). Except for *U. maydis*, most tested fungal species possessed a homolog of GLT-1 and one or two HGTs. For example, several functionally identified low- and high-affinity glucose transporters (MstE, MstC/HxtE, and HxtC in *A. nidulans* [60–62]; MstE, MstA, and MstH in *A. niger* [63, 64]; and CgHXT5, CgHXT3, and CgHXT1 in *C. graminicola* [65]) were clustered into the respective GLT-1, HGT-1, and HGT-2 groups (Fig. 11), suggesting robust conservation of this dual-affinity transport system in the kingdom Fungi. Although *S. cerevisiae* is widely accepted to possess a glucose dual-affinity transport system (*K*_m_ = ~20 mM for the low-affinity system and ~1 mM for the high-affinity system), the affinity difference between the two systems in yeast is not so extreme as in filamentous fungi such as *N. crassa*, reflecting their different evolutionary trajectories shaped by different growth niches in nature.

## Discussion

### Dual-affinity systems for nutrient transport in filamentous fungi: revision of an old story

**Fig. 12:**
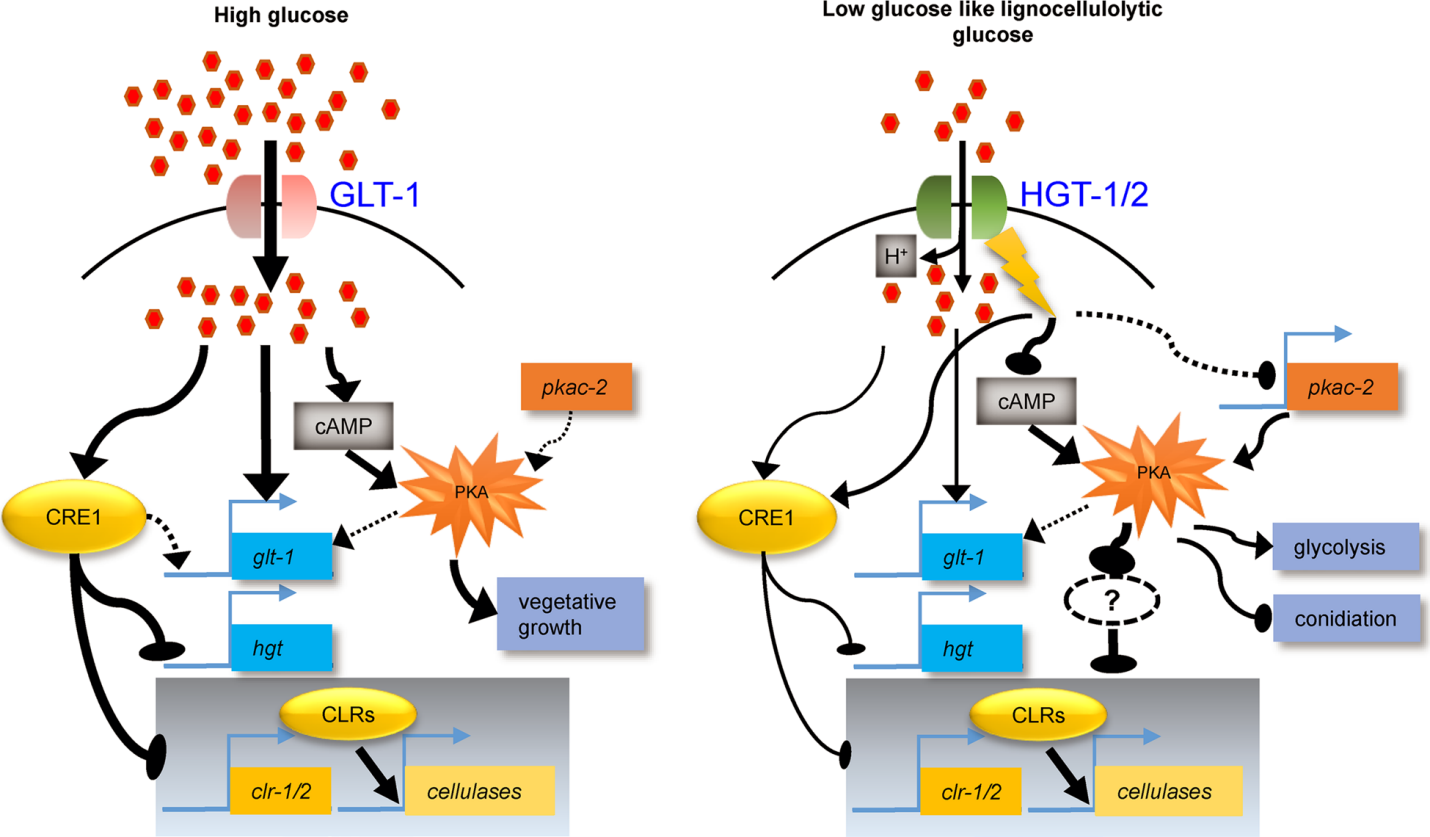
Working model of how the glucose dual-affinity transport system functions in glucose transport, signaling, and CCR. Under high levels of glucose, *N. crassa* predominantly uses the low-affinity, but high-capacity GLT-1 protein for nutrient assimilation. Adequate glucose stimulates CRE-1-mediated CCR to repress cellulase expression for non-preferred carbon (e.g., plant cell walls) utilization, whereas the cAMP-PKA pathway is induced for vegetative growth. When external glucose is depleted or limited, however, HGT-1/-2 are rapidly derepressed by the lifting of CCR and transport the limited external glucose. This process synergistically downregulates the cAMP-PKA pathway via one or more mechanisms to decrease cAMP levels and *pkac*-*2* expression, thus leading to attenuated glycolytic activity. Constant exposure of cells to glucose-limited conditions leads to sexual sporulation for survival, thereby also resulting in downregulation of *clr*-*1/2* and cellulase expression, and reactivation of CCR on cellulose. Whether cAMP-PKA can regulate CRE-1 and/or CLR-1/-2 directly or through downstream transcriptional factors to function in plant cell wall deconstruction requires further investigation

In individual organisms, uptake affinities usually vary widely among different nutrients [8, 10, 14, 66–69]. In filamentous fungi such as *A. niger* and *N. crassa*, glucose uptake at the cellular level behaves in a dual-transport fashion [8–10, 70]. Several physiological characteristics of the glucose dual-transporter system in *N. crassa* are worth noting: (1) the low- and high-affinity systems (systems I and II) cover a 1000-fold difference in affinity making this system a good representative model of glucose uptake; (2) system I is a glucose diffusion system, while system II is an active, H^+^-co-transport mechanism; and (3) system I is constantly expressed at high glucose levels, whereas system II is subject to repression by glucose and can be de novo synthesized under low- or no-carbon conditions [8–10, 23, 24, 71]. The dual-affinity glucose transport system in *N. crassa* has been known about for nearly 50 years since the 1970s but has not been investigated systematically at the gene level until the present study, although some characteristics of *hgt*-*1* and GLT-1 have been identified before [33, 40]. Here, three genes were assigned to this dual-system: GLT-1 forms the low-affinity transport system that takes up glucose under adequate glucose conditions, whereas HGT-1 and HGT-2 comprise the high-affinity system that imports sugar when the external glucose concentration is very low (Fig. 12). Whether this dual-affinity transport system, which is highly conserved across fungal species (Fig. 11), increases the adaptability of fungal systems in nature needs to be further investigated. Moreover, because both sensing and import are pivotal for microorganism growth [72], the finding that HGT-1/-2 perform glucose signaling in addition to their transport functions suggests a novel role for HGT-1/-2 as sensor-like transporters (the so-called “transceptors”) in the dual-affinity transport system, thereby promoting both cell growth and interaction with the environment. Together, the findings here, to our knowledge, represent the first genome-wide molecular characterization of the dual-affinity glucose transport system in filamentous fungi.

### Low- vs. high-affinity systems: a trade-off between transport capacity and gene dosage

Despite the apparent exclusiveness of high-affinity and high-capacity, high-affinity transporters, such as *hgt*-*1/*-*2*, are always highly expressed in derepressed conditions (Fig. 2 and Additional file 5: Figure S3). Even though the low-affinity system I gene *glt*-*1* displayed considerably higher expression than other sugar transporters in the presence of high glucose (Fig. 2), its mRNA level was not at the same order of magnitude as those of *hgt*-*1/*-*2* under low-glucose conditions (e.g., RPKM = 210 for *glt*-*1* vs. 5,700 for *hgt*-*1*; Fig. 2b). In contrast, the *V*_max_ value of GLT-1 was considerably higher than those of HGT-1/-2 (30.75 ± 1.34 mmol h^−1^ g^−1^ DCW for GLT-1 vs. 26.42 ± 0.38 and 78.11 ± 4.46 µmol h^−1^ g^−1^ DCW for HGT-1 and -2). This suggests that the high-affinity transport system, consisting of low-capacity transporters, compensates for this inherent kinetic handicap by increasing the number of individual proteins to keep pace with the low-affinity system in uptake velocity. The maximum glucose uptake rate of the derepressible system II was demonstrated to be similar to that of the glucose-inducible system I [8]. The highly expressed high-affinity transport system is therefore able to maintain nutrient homeostasis while external nutrients are being depleted, thereby providing sufficient preparation time for starvation and consequent cell recovery when nutrients are replenished [68].

### Glucose transport by the derepressible glucose transporters HGT-1/-2 suppresses lignocellulase gene expression and vegetative growth under carbon-limited environments, thereby favoring fungal sporulation for survival

In a number of filamentous fungi, deletion of *cre*-*1*/*creA*/*cre1* promotes the utilization of alternative carbon sources, particularly through derepression of cellulolytic enzymes [4, 73–75]. Although deletion of *cre*-*1* in the Δ*2hgt* strain resulted in faster growth on Avicel (Fig. 7), simply concluding that the hyper-production of cellulases in Δ*2hgt* is related to the regulator CRE-1 is not warranted: single deletion of *cre*-*1* only resulted in approximately 30% higher protein production than in the WT [4], far less than the 180–210% increases are seen in the Δ*2hgt* and Δ*2hgt*;Δ*cre*-*1* mutants (Fig. 7a). HGT-1/-2 appear to connect extracellular glucose signaling with the internal CCR signal, because the Δ*2hgt* mutant displayed moderate resistance to 2-DG inhibition (Fig. 6d, e). This HGT-mediated suppression is partly associated with CRE-1-mediated CCR, as downregulation of *cre*-*1* in Δ*2hgt* occurred in the late phase of cellulose utilization (Fig. 6a; Additional file 10: Table S4). Despite these findings, exactly how HGT-1/-2 function with CRE-1 (or other unknown factors) to mediate CCR in *N. crassa* remains to be elucidated. Intriguingly, the transcriptional regulator *vib*-*1*, which was recently found to coordinate glucose signaling and CCR during plant cell wall degradation [5], was not strongly upregulated when *hgt*-*1/*-*2* were deleted (Additional file 10: Table S4). Future investigations of the regulatory network consisting of VIB-1-mediated CCR and the HGT-mediated glucose response should help to decipher fungal glucose signaling and CCR regulation.

In *S. cerevisiae*, extracellular glucose can be sensed by two membrane transporter-like sensors, Rgt2p and Snf3p [11]. These two sensors possess a long cytoplasmic C-terminus harboring one or two short conserved sequence blocks that are essential for glucose signal transduction [12]. The identical homolog of Rgt2p/Snf3p in *N. crassa* is RCO-3 (Fig. 11), which also contains an extremely long C-terminus in the cytoplasm (Fig. 10a). Mutation of *rco*-*3* in *N. crassa* leads to severe cell defects in glucose uptake as well as decreased growth in the presence of high glucose levels, but the ability to sense low levels of glucose is probably still retained [22]. These results were corroborated in our study: qRT-PCR analysis revealed that *glt*-*1* was dramatically downregulated (~100-fold) in Δ*rco*-*3* compared with the WT, resulting in synergistically elevated expression (ten- to 30-fold) of *hgt*-*1/*-*2* and NCU06358 (Additional file 14: Figure S9). Because the derepression of high-affinity glucose transporters *hgt*-*1/*-*2* was not affected in the Δ*rco*-*3* mutant, RCO-3 might act as a low-affinity glucose sensor [22], whereas HGT-1/-2 are high-affinity ones.

Intriguingly, glycolysis and fermentation were stimulated in Δ*2hgt* (Additional file 11: Table S5). This phenomenon is quite similar to the “Warburg effect” in cancer cells, where glycolysis is improperly activated by several important kinases, such as PI3K/AKT and tyrosine kinase [76]. Similarly, the cAMP-PKA signaling pathway cannot be excluded as a possible explanation for these HGT-dependent functions (Fig. 12): (1) the internal cAMP level was significantly higher in the Δ*2hgt* mutant during cellulose utilization (Fig. 9); (2) *pkac*-*2*, one of only two PKA catalytic subunits reported to date in *N. crassa* [57], was strongly and constantly upregulated (Additional file 11: Table S5); and (3) asexual conidiation-related genes were extensively inactivated in Δ*2hgt* (Additional file 11: Table S5). In *N. crassa*, inactivation of PKA leads to strong derepression of submerged-culture conidiation as well as aerial sporulation [57, 77]. Future investigations of the connection between HGT and the cAMP-PKA signaling pathway as well as the specific roles of HGT in glycolysis, plant cell wall deconstruction, and sporulation will help us to understand the architecture of nutrient signaling regulation in filamentous fungi (Fig. 12).

Sporulation and quiescence are conserved strategies for survival when organisms encounter nutrient depletion or other chemical/physical stresses [78, 79]. In this regard, signaling to suppress vegetative growth and activate sporulation is a preferred mechanism for fungi to overcome severe growth environments. As revealed by this study, HGTs are strongly derepressed under carbon-limited or lignocellulolytic conditions, which lead to the inactivation of glycolysis but activation of asexual sporulation (Fig. 12). HGT-mediated repression of cellulosic growth therefore favors fungal conidiation, which might be beneficial for species survival under carbon-limited environments. Conversely, upon encountering plentiful glucose, it should be noted that *N. crassa* can inhibit system II [23] and synergistically elevate the expression of the low-affinity transporter *glt*-*1* for cell proliferation (Fig. 12).

## Conclusions

In this study, HGT-1/-2 and GLT-1 were identified as major derepressible and glucose-inducible components, respectively, of the high- and low-affinity glucose transport systems in *N. crassa*. Growth defects due to loss of *glt*-*1* were restored by upregulation of the high-affinity transporters *hgt*-*1/*-*2*. In addition to their glucose-transporting functions under carbon-limited or cellulolytic conditions, HGT-1/-2 also mediate glucose signaling to connect extracellular nutrient availability with internal catabolite repression and metabolism, and thus may act as glucose transceptors. Simultaneous deletion of *hgt*-*1* and *hgt*-*2* leads to comprehensive derepression of a large group of genes including those encoding glycolysis enzymes and plant cell wall-degrading enzymes, which are associated with CRE-1-related CCR, transcriptional regulation by CLR-1/-2, and the cAMP-PKA signaling pathway. Given the wide conservation of these dual-affinity transport components across the fungal kingdom, future investigations of GLT-1 and HGT-1/-2, and studies of HGT-mediated signaling in other fungal systems will shed new light on long-standing questions about the physiological roles and evolutionary traits of the dual-affinity transport system, and thus inform studies on fungal glucose signaling, pathogenicity, and plant cell wall deconstruction for biorefinery.

## Methods

### Strains, media, and culture conditions

The *N. crassa* WT strain (FGSC2489) and mutants Δ*glt*-*1* (FGSC13161), Δ*hgt*-*1* (FGSC22819), Δ*hgt*-*2* (FGSC18807), Δ*NCU05897* (FGSC13717), Δ*NCU00450* (FGSC15906), and Δ*rco*-*3* (FGSC17928) were obtained from the Fungal Genetics Stock Center (FGSC) [80]. The mutant Δ*cre*-*1* was a gift from the laboratory of Professor N. Louise Glass of the University of California, Berkeley. Multiple deletion mutants were generated via sexual crosses of the above-mentioned mutants (FGSC protocol, http://www.fgsc.net/Neurospora/NeurosporaProtocolGuide.htm). Artificial misexpression strains were constructed by transforming the Δ*2hgt*;*his*-*3* ^−^ strain with the linearized plasmid pMF272 harboring the clock-controlled gene 1 (*ccg*-*1*) or *M. grisea* ribosomal protein 27 (*MgRP27*) [44, 48] promoters, various gene-coding sequences or point-mutated analogs, and flanking regions from the *his*-*3* gene sequence [81]. Transformants with histidine prototrophy were selected for further purification via microconidial separation (FGSC protocol, http://www.fgsc.net/Neurospora/NeurosporaProtocolGuide.htm). At least three purified biological transformants per selected gene were used for downstream experiments. *N. crassa* strains were pre-grown on slants containing 3 mL Vogel’s minimal medium [82] with 2.0% (*w/v*) sucrose as a sole carbon source for 1–2 days in darkness at 28 °C, followed by constant light for 6–8 days at room temperature to obtain mature conidia. Unless indicated, *N. crassa* conidia were inoculated into liquid Vogel’s minimal medium with various carbon sources at 10^6^ conidial mL^−1^ with constant light shaking at 200 rpm, 25 °C. For CCR sensitivity analysis, 2-DG (Sigma-Aldrich, St. Louis, MO, USA) was added to 100 mL liquid Vogel’s minimal medium containing 2.0% Avicel PH-101 (Sigma-Aldrich) to a final concentration of 0.1%.

For media shift experiments, *N. crassa* cultures were pre-grown on 100 mL Vogel’s salts supplemented with 2.0% sucrose for 16 h, with the generated mycelia then filtered through six layers of gauze and immediately washed with sterilized water at least five times. The mycelia from each flask pre-culture were then transferred to 100 mL Vogel’s medium containing 0–10% glucose as the sole carbon source, followed by 60-min growth according to previous studies [23]. The mycelia were subsequently sampled via filtration and flash frozen in liquid nitrogen for total RNA extraction. For low-glucose uptake assays, the washed mycelia were transferred to 100 mL carbon-free Vogel’s medium with shaking for 90 min [23]. The carbon-starved mycelia were then centrifuged at 3220×*g* for 5 min, and approximately 4 mL wet mycelia were transferred into new medium containing 100 mL Vogel’s salts plus 0.02% glucose with shaking at 25 °C and 200 rpm. Culture supernatants (500 µL) were taken at indicated time points (0, 5, 10, 20, and 30 min) and immediately filtered through 0.22-µm membranes. Each mycelial culture used for the glucose uptake assay was harvested, dried, and weighed. Glucose concentrations were measured with a Megazyme d-Glucose Assay kit according to the manufacturer’s instructions (Megazyme, Wicklow, Ireland). Equivalent glucose uptake was defined as the amount of glucose consumed per gram of dried mycelia.

*Saccharomyces cerevisiae* EBY.VW4000 [41], a gift from Professor Eckhard Boles, was grown in YPM medium (1.0% yeast extract, 2.0% peptone, 2.0% maltose, and optionally 2.0% agar) for subsequent use. Recombinant strains were cultured in synthetic complete medium (SC) supplemented with drop-out amino acids lacking uracil (Ura^−^) and 2.0% glucose or maltose as the sole carbon source (with or without 2.0% agar). For the growth complementation assay, recombinant strains were incubated in liquid SC (Ura^−^) medium with maltose. Cells were harvested at an optical density at 600 nm (OD_600_) of 1.0–2.0, washed twice with distilled water, and resuspended in distilled water to an OD_600_ of 0.25–0.30. Serially diluted cells (×1, ×10, and ×100; 2 µL for each diluted clone) were plated on solid SC (Ura^−^) medium containing either maltose or glucose. Growth on maltose or glucose medium at 3 or 6 days, respectively, was photographed with a Canon camera (Canon EOS207, Japan).

### Plasmid construction

The open reading frames (ORFs) of *cre*-*1*, *hgt*-*1/*-*2*, and *glt*-*1* were amplified from the cDNAs of *N. crassa*. For misexpression of these genes in the Δ*2hgt* mutant, their ORFs were inserted into the multiple cloning sites of the shuttle vector pMF272 [83] driven by either the *ccg*-*1* or the *MgRP27* promoter and tagged with enhanced green fluorescence protein (eGFP). The analogs *hgt*-*1*(R172K), *hgt*-*2*(R155K), and *glt*-*1*(R167K) were generated by site-directed mutagenesis using high-fidelity PCR polymerase (Thermo Fisher Scientific, Waltham, MA, USA). For heterologous expression in *S. cerevisiae*, these transporter ORFs were recombined with the yeast shuttle vector pRS426 [84] fused with the native phosphoglycerate kinase-1 (*pgk*-*1*) promoter and the eGFP reporter gene. Cloning primers are listed in Additional file 15: Table S6. Restriction enzymes were purchased from Thermo Fisher Scientific. All recombinant plasmids were amplified in *Escherichia coli* strain DH5α and sequenced for gene authenticity.

### RNA extraction, sequencing, and data analysis

Mycelial sampling and total RNA extraction were performed as described in a previous study [43]. Briefly, cultured mycelia were harvested by filtration and immediately frozen in liquid nitrogen. Total RNA was isolated with Trizol reagent (Invitrogen, Carlsbad, CA, USA) and further purified using DNase I (RNeasy Mini kit, Qiagen, Hilden, Germany). The RNA concentration and OD_260_/OD_280_ were measured with a Nanodrop 2000c (Thermo Scientific), and RNA integrity was checked by agarose gel electrophoresis and using an Agilent 2100 (Agilent Technologies, Santa Clara, CA, USA).

Qualified RNA with an OD_260_/OD_280_ >1.8 and RIN (RAN Integrity Number) >7.0 was prepared according to Shenzhen BGI (Shenzhen, China) and Novogene (Tianjin, China) standard protocols, and sequenced on an Illumina HiSeq 2000/2500 platform (San Diego, CA, USA). The biological reproducibility of the RNA-Seq data was demonstrated to be high through sample-to-sample clustering [25] and Spearman correlation analysis (Additional file 1: Figure S1, Additional file 9: Figure S6). Read mapping and counting using TopHat2 (version 2.0.12) [85] and HTSeq (version 0.6.0) [86] was performed as previously described [43]. The reads mapped to each transcript were used to calculate normalized transcript abundance (as RPKM [87]) and to perform differential gene expression analysis in GFOLD (version 1.1.0) [88] and DESeq2 (version 1.2.10) [25]. Genes with a DESeq2 *P* value <1 × 10^−4^ were considered to be statistically differentially expressed at a robust significance level. Because genes with |GFOLD| > 1 are empirically more likely to be of biological importance [88], a combined criterion of |GFOLD| > 1 and *P* < 1 × 10^−4^ was applied for genome-wide differential gene analysis. DESeq2 [25] was used to estimate expression fold-changes, which represent apparent expression changes that may also be useful for assessment of biological expression variations. RNA-Seq raw data are available at the Gene Expression Omnibus under accession number GSE78952.

Hierarchical clustering analysis was performed using Cluster 3.0 [89] or the pheatmap package in R (version 1.0.2) (http://www.r-projectorg). To generate a clustering heatmap, the RPKM values of each gene were log-transformed and calculated by the complete linkage method with Euclidean distance as the similarity metric. Selected differentially expressed genes were submitted to the MIPS Functional Category Database [28], and significantly enriched pathways were estimated.

### qRT-PCR

Quantitative real-time reverse-transcription polymerase chain reaction was performed using SYBR Green Realtime PCR master mix (Toyobo, Osaka, Japan) according to the manufacturer’s instructions on a CFX96 real-time PCR detection system (Bio-Rad, Hercules, USA). Each reaction was conducted in duplicate or triplicate. The actin gene (NCU04173) was used as an endogenous control for all experiments. All primers used in this study are listed in Additional file 15: Table S6. The relative expression level of each gene was calculated using the 2^−ΔΔCt^ method [90].

### cAMP measurements

Samples submerged in Vogel’s medium with 2.0% Avicel and cultured for the indicated time periods were harvested by filtration and immediately frozen in liquid nitrogen. Mycelia were finely ground in liquid nitrogen, transferred to 1.0% hydrochloric acid, briefly vortexed, and frozen at −80 °C until use. Before cAMP measurements, the samples were thawed at 4 °C and centrifuged at 12,000×*g* for 15 min at 4 °C. The supernatant was used for the cAMP assay following the Applied Biosystems (Waltham, MA, USA) protocol. The protein concentration of each supernatant was quantified by the Bradford method (Bio-Rad). The protein in the pellet was solubilized in 0.5% sodium dodecyl sulfate plus 0.1 M sodium hydroxide, vortexed for 30 s, and also measured using the Bradford method.

### Secreted enzyme and dried mycelial weight assays

For secreted protein assays, 800 µL of each culture supernatant was collected during the growth period (2–7 days with Avicel and 1–6 days with cellobiose), centrifuged at 15,294×*g* for 8 min to remove mycelia, and stored at 4 °C for analysis within 24 h or at −20 °C for sodium dodecyl sulfate polyacrylamide gel electrophoresis (Novex NuPAGE Pre-cast Protein Gels; Thermo Fisher Scientific).

The total secreted protein content was determined using the Bradford method (Bio-Rad) with bovine serum albumin as a standard. Carboxymethyl cellulase and xylanase activities were measured using an Azo-CMC/xylan kit (Megazyme) according to the manufacturer’s instructions. Exoglucanase activity was assayed as previously described using *p*-nitrophenyl-d-cellobioside (Sigma-Aldrich) as the substrate [43].

Mycelia grown on sucrose, glucose, and cellobiose for designated times were harvested, dried, and weighed. Biomass dry weights of the Avicel cultures were measured according to a previous study [43] with a slight modification. In brief, 5 mL of thoroughly mixed culture broth was centrifuged at 3220×*g* for 5 min. After discarding the supernatant, 3 mL 80% (*v/v*) acetic acid:concentrated nitric acid (10:1, *v/v*) reagent was added and the mixture was boiled in water for 1 h to solubilize the fungal biomass. This procedure was repeated with a fresh 3-mL aliquot of acetic acid:nitric acid reagent. The reaction mixture (residual Avicel) was then centrifuged, dried, and weighed. Mycelial dry weight was defined as the dry weight of the original 5-mL culture minus that of the reaction mixture.

### Microscopy and imaging

Visualization of eGFP-tagged glucose transporters in *S. cerevisiae* and *N. crassa* was conducted using a 100 × 1.4 NA oil immersion objective on a Leica TCS SP5 II laser scanning confocal microscope (Leica, Wetzlar, Germany). For confocal microscopy of yeast, single clones of recombinant EBY.VW4000 strains harboring various glucose transporters were inoculated into SC(Ura^−^) liquid medium supplemented with 2.0% maltose and grown overnight to an OD_600_ of 1.0–2.0. The cell cultures were centrifuged, washed twice with sterile water, and resuspended in phosphate-buffered saline solution. For microscopy of *N. crassa*, recombinant *N. crassa* was pre-grown on Vogel’s medium with 2.0% sucrose for 16 h and then briefly washed with sterilized water at least five times and resuspended in Vogel’s medium with either 2.0% Avicel, glucose, or no added carbon for 4 h with shaking at 200 rpm and 25 °C. Before confocal scanning, mycelia were treated with 1 µg mL^−1^ 4′,6-diamidino-2-phenylindole for 15 min as needed. Images were processed using the Leica Microsystems LAS AF-TCS MP (version 2.4.1) and ImageJ (version 1.47) software.

### Radiolabeled glucose transport

A glucose transport assay in yeast was performed according to a previously published protocol [33].

### Phylogenetic analysis

Amino acid sequences of GLT-1 and HGT-1/-2 (http://www.broadinstitute.org) were used as queries in BLASTp searches against the protein sequences in the genomes of *A. nidulans*, *A. niger*, *A. oryzae*, *A. fumigatus*, *M. thermophile*, *T. reesei*, *B. cinerea*, *C. globosum*, *C. graminicola*, *F. graminearum*, *M. oryzae*, *U. maydis*, and *T. marneffei* in the National Center for Biotechnology Information (NCBI) and DOE Joint Genome Institute (JGI) databases. The best hits with identity >50%, *E*-value <1 × 10^−10^, and coverage >60% were selected as the closest homologs of GLT-1 and HGT-1/-2, with the exception of two GLT-1 homologs (Um XP_758184.1: 43% identity; Mo XP_003719105.1: 40% identity). Among these highly conserved homologs, under-documented low- and high-affinity glucose transporters served as positive controls. Hexose transporters of *S. cerevisiae* and *H. sapiens*, and reported pure sensors in *S. cerevisiae* and *N. crassa* were included as outgroups. The protein sequences of these MFS transmembrane proteins were aligned with ClustalW [91] using the following parameters: protein weight matrix, Gonnet; gap opening penalty, 10; gap extension, 0.2; and gap distance, 5. A phylogenetic tree was constructed under the JTT amino acid substitution model by maximum likelihood in MEGA 5.2 [92], with 1000 bootstrap replicates.

### Data plotting

All figures were plotted on the R program platform (http://www.r-project.org/) or Excel 2013. Unless otherwise indicated, values represent the means of at least three replicates; error bars show standard deviations.

## Additional files

**Additional file 1: Figure S1.** Validation of RNA-Seq data of *N. crassa* in response to a glucose gradient. (**a**) Sample-to-sample clustering and Spearman analysis. (**b**) No-carbon data reproducibility between published data [26] and this study.

**Additional file 2: Table S1.** Gene expression profiling and differential analysis of transcriptional responses to a glucose gradient in *N. crassa*. For each gene, RPKM, log_2_ fold change, and *P* values are given for each level of glucose *vs*. 2.0% glucose.

**Additional file 3: Table S2.** Functional category analysis (FunCat) of up/downregulated genes at each level of glucose *vs.* 2.0% glucose. Sheet 1: functional category analysis of upregulated genes; sheet 2: functional category analysis of downregulated genes; sheet 3: gene expression dynamics and annotation of pioneer responses to glucose depletion (no-carbon or glc_0.05% *vs*. glc_2.0%).

**Additional file 4: Figure S2.** Transient glucose uptake of WT, Δ*2hgt*, Δ*2hgt*;Δ*NCU00450*, and Δ*2hgt*;Δ*NCU05897* strains. Values represent means of triplicates; error bars show standard deviations.

**Additional file 5: Figure S3.** Heatmap of expression modes of *glt-1* and *hgt-1/-2* on various carbon sources. Log-transformed RPKM values of *glt-1* and *hgt-1/-2* were clustered by pheatmap.

**Additional file 6: Figure S4.** Secreted protein production of Avicel cultures of WT, Δ*hgt-1*, Δ*hgt-2*, and Δ*2hgt* strains. Each data point represents the mean of triplicates; error bars indicate standard deviations.

**Additional file 7: Figure S5.** Relative dried mycelial weights of Δ*glt-1*, Δ*2hgt*, and Δ*2hgt*;Δ*glt-1* strains *vs.* the WT grown on Avicel for the indicated times. Average values and standard deviations from at least three replicates are shown.

**Additional file 8: Table S3.** Relative enzyme activity per dried cell weight (gram) *vs.* WT and Student’s *t*-test of Figure 5C.

**Additional file 9: Figure S6.** Validation of RNA-Seq data of the WT, Δ*cre-1*, Δ*2hgt*, and Δ*2hgt*;Δ*cre-1* grown on Avicel for the indicated times. The results of sample-to-sample clustering and Spearman analysis are shown.

**Additional file 10: Table S4.** Gene expression profiling and differential expression analysis of Δ*2hgt* and Δ*2hgt*;Δ*cre-1* vs. the WT or Δ*cre-1* grown on Avicel for the indicated times.

**Additional file 11: Table S5.** Hierarchical clustering and functional category analysis of differentially expressed genes in Δ*2hgt vs.* the WT. Sheet 1: expression clustering and functional annotation of the 629 genes upregulated in Δ*2hgt* vs. the WT; sheet 2: functional category analysis of genes up/downregulated in Δ*2hgt* vs. the WT; sheet 3: expression profile and functional annotation of the major documented genes in the conidiation process of *N. crassa*.

**Additional file 12: Figure S7.** Phenotypes of WT, Δ*glt-1*, Δ*cre-1*, Δ*2hgt*, Δ*2hgt*;Δ*glt-1*, Δ*2hgt*;Δ*cre-1*, and Δ*2hgt*;Δ*glt-1*;Δ*cre-1* strains grown on 2.0% cellobiose as the sole carbon source. (A) Dynamics of secreted protein production by the tested strains. (B) Endo-glucanase (CMCase), xylanase, and exo-glucanase (pNPCase) of 7-day-culture supernatants. (C) Residual glucose and (D) cellobiose of the supernatants. (E) Dried mycelial weights of tested strains grown for 1 and 3 days. Values represent averages of triplicates; error bars show standard deviations.

**Additional file 13: Figure S8.** Confocal imaging of the Δ*2hgt* mutant expressing CRE-1 controlled by *ccg-1* or ribosomal protein 27 promoters after 16 h of mycelial pre-growth under no-carbon, Avicel, and glucose conditions, respectively. The scale bar corresponds to 10 µm.

**Additional file 14: Figure S9.** Relative expression levels of *glt-1*, *hgt-1/-2*, and another putative glucose transporter, NCU06358, in the Δ*rco-3* mutant *vs.* the WT treated with 0.5% glucose for 1 h. Average values and standard deviations of two biological replicates/two technical replicates are shown.

**Additional file 15: Table S6.** Primers used in this study.

## Abbreviations

cAMP: cyclic adenosine monophosphate
CAZy: carbohydrate-active enzyme
CCR: carbon catabolite repression
CMCase: carboxymethyl cellulase
eGFP: enhanced green fluorescence protein
FGSC: Fungal Genetics Stock Center
GEO: gene expression omnibus
MFS: major facilitator superfamily
PKA: protein kinase A
qRT-PCR: quantitative real-time reverse transcription polymerase chain reaction
RPKM: reads per kilobase per million mapped reads
SDS-PAGE: sodium dodecyl sulfate polyacrylamide gel electrophoresis
WT: wild type
