# Supplementary figures and images for: Identification and characterization of the glucose dual-affinity transport system in Neurospora crassa: pleiotropic roles in nutrient transport, signaling, and carbon catabolite repression
Source: Biotechnol Biofuels. 2017 Jan 19;10:17. doi: 10.1186/s13068-017-0705-4 (PMC5244594; doi:10.1186/s13068-017-0705-4)

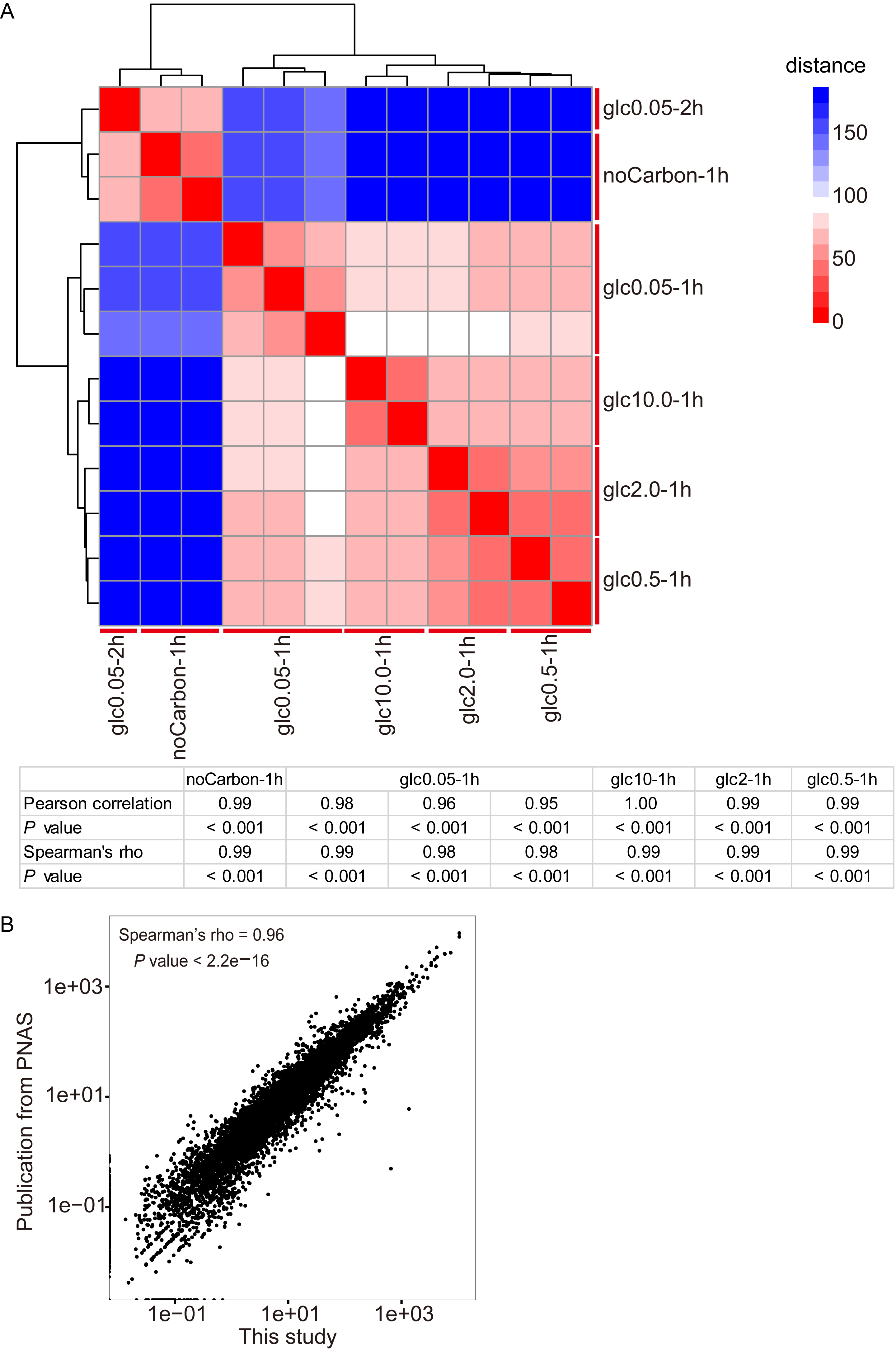

Supplement: Supplementary file 1 — Additional file 1: Figure S1. Validation of RNA-Seq data of N. crassa in response to a glucose gradient. (a) Sample-to-sample clustering and Spearman analysis. (b) No-carbon data reproducibility between published data [26] and this study. [file 13068_2017_705_MOESM1_ESM.tiff]

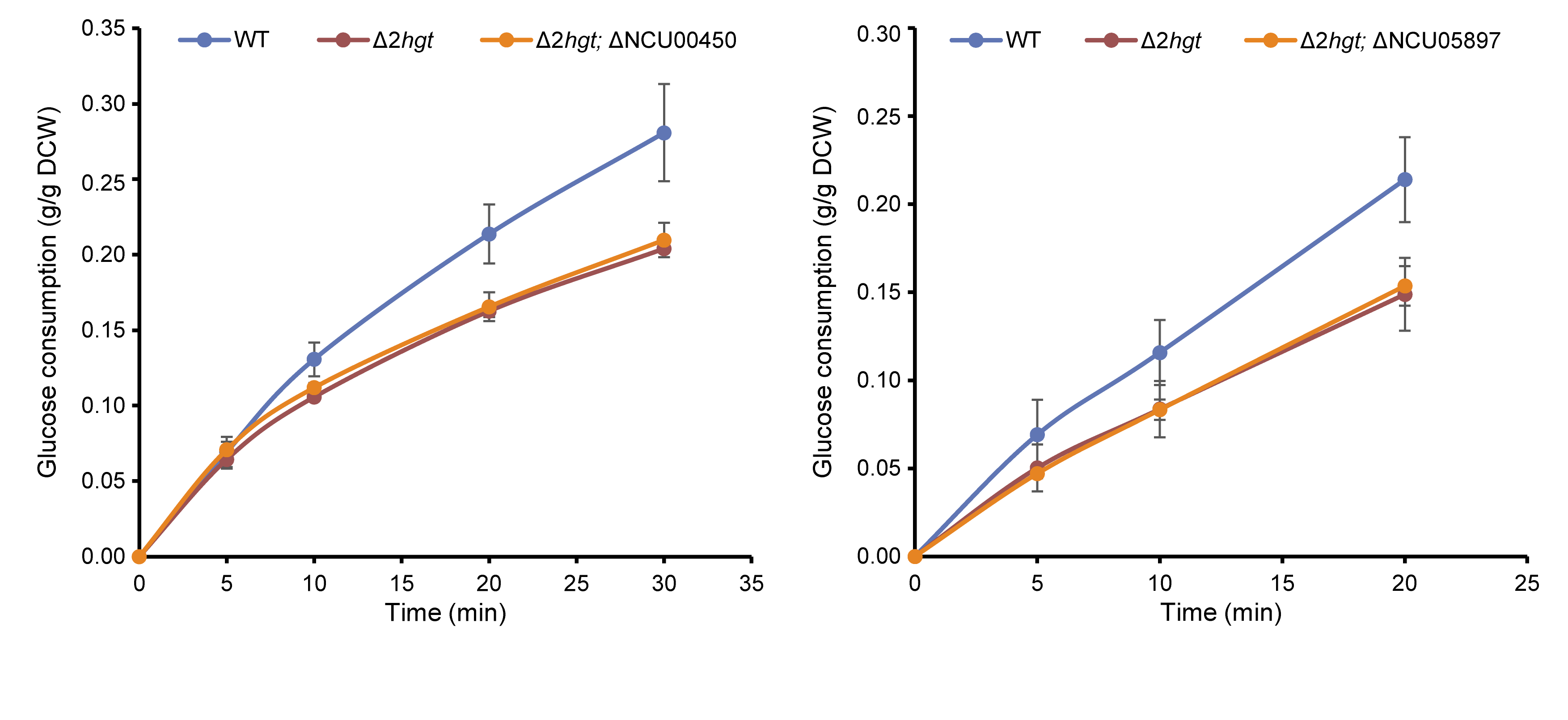

Supplement: Supplementary file 4 — Additional file 4: Figure S2. Transient glucose uptake of WT, Δ2hgt, Δ2hgt;ΔNCU00450, and Δ2hgt;ΔNCU05897 strains. Values represent means of triplicates; error bars show standard deviations. [file 13068_2017_705_MOESM4_ESM.tiff]

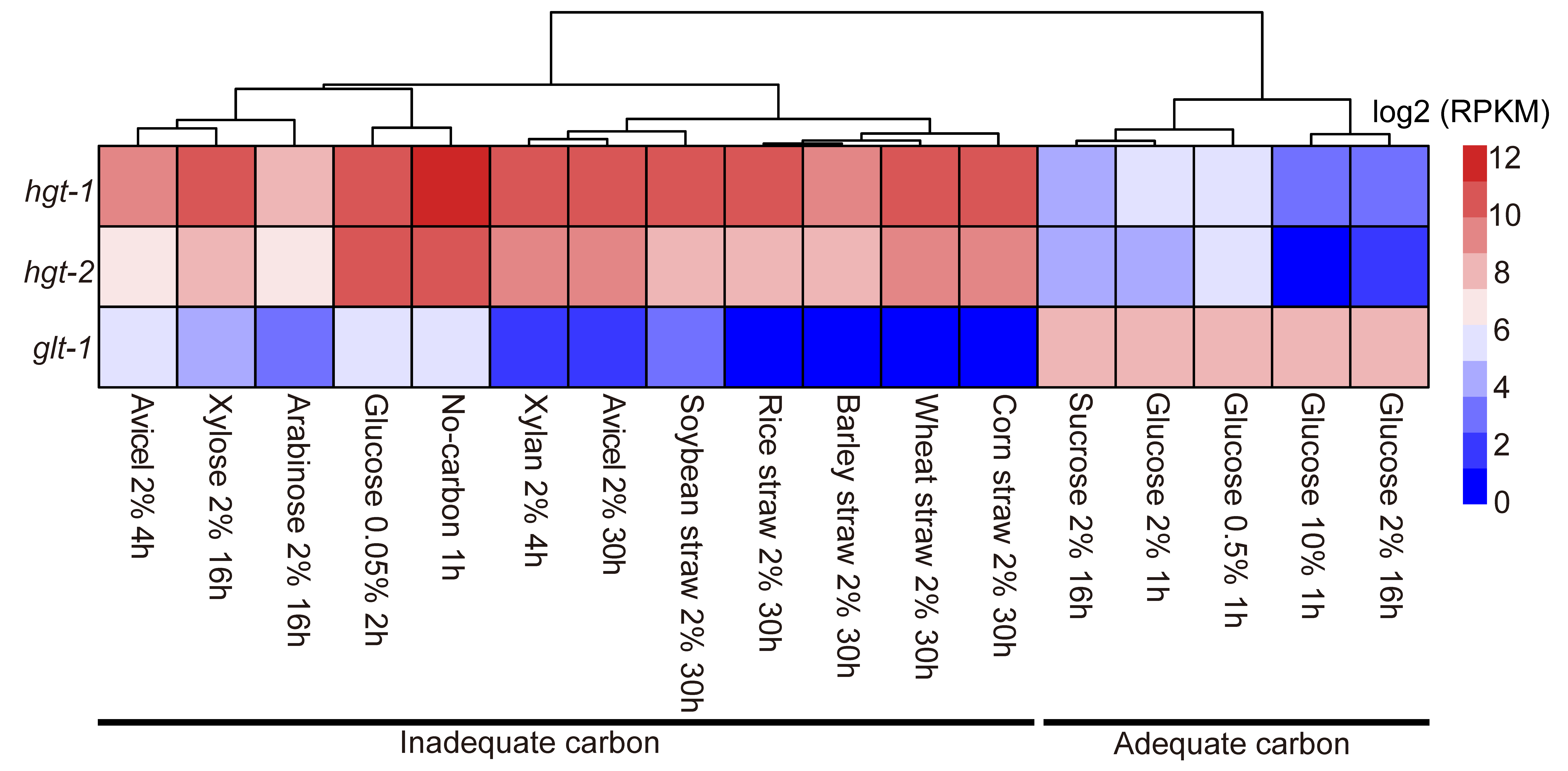

Supplement: Supplementary file 5 — Additional file 5: Figure S3. Heatmap of expression modes of glt-1 and hgt-1/-2 on various carbon sources. Log-transformed RPKM values of glt-1 and hgt-1/-2 were clustered by pheatmap. [file 13068_2017_705_MOESM5_ESM.tiff]

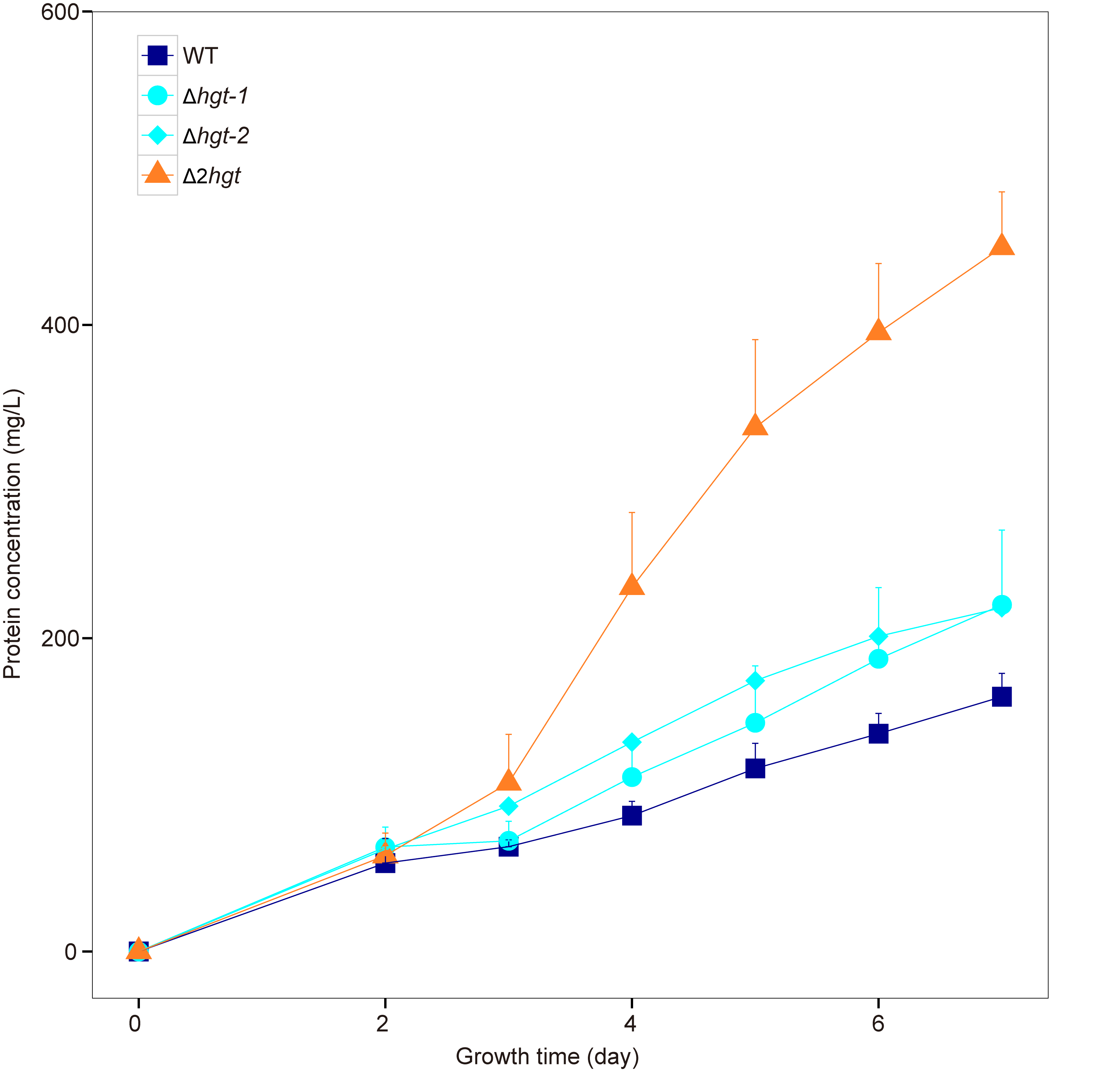

Supplement: Supplementary file 6 — Additional file 6: Figure S4. Secreted protein production of Avicel cultures of WT, Δhgt-1, Δhgt-2, and Δ2hgt strains. Each data point represents the mean of triplicates; error bars indicate standard deviations. [file 13068_2017_705_MOESM6_ESM.tiff]

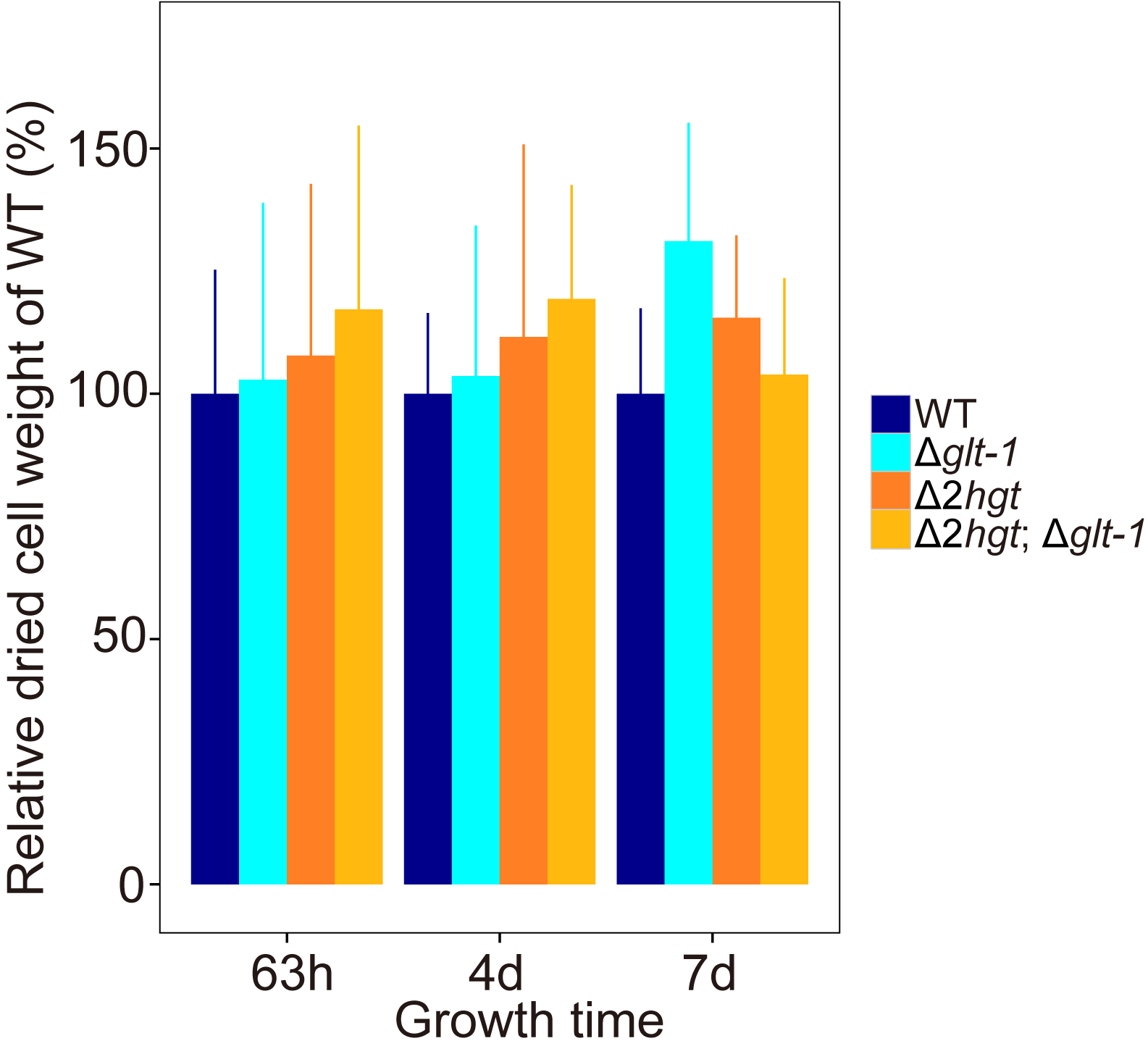

Supplement: Supplementary file 7 — Additional file 7: Figure S5. Relative dried mycelial weights of Δglt-1, Δ2hgt, and Δ2hgt;Δglt-1 strains vs. the WT grown on Avicel for the indicated times. Average values and standard deviations from at least three replicates are shown. [file 13068_2017_705_MOESM7_ESM.tiff]

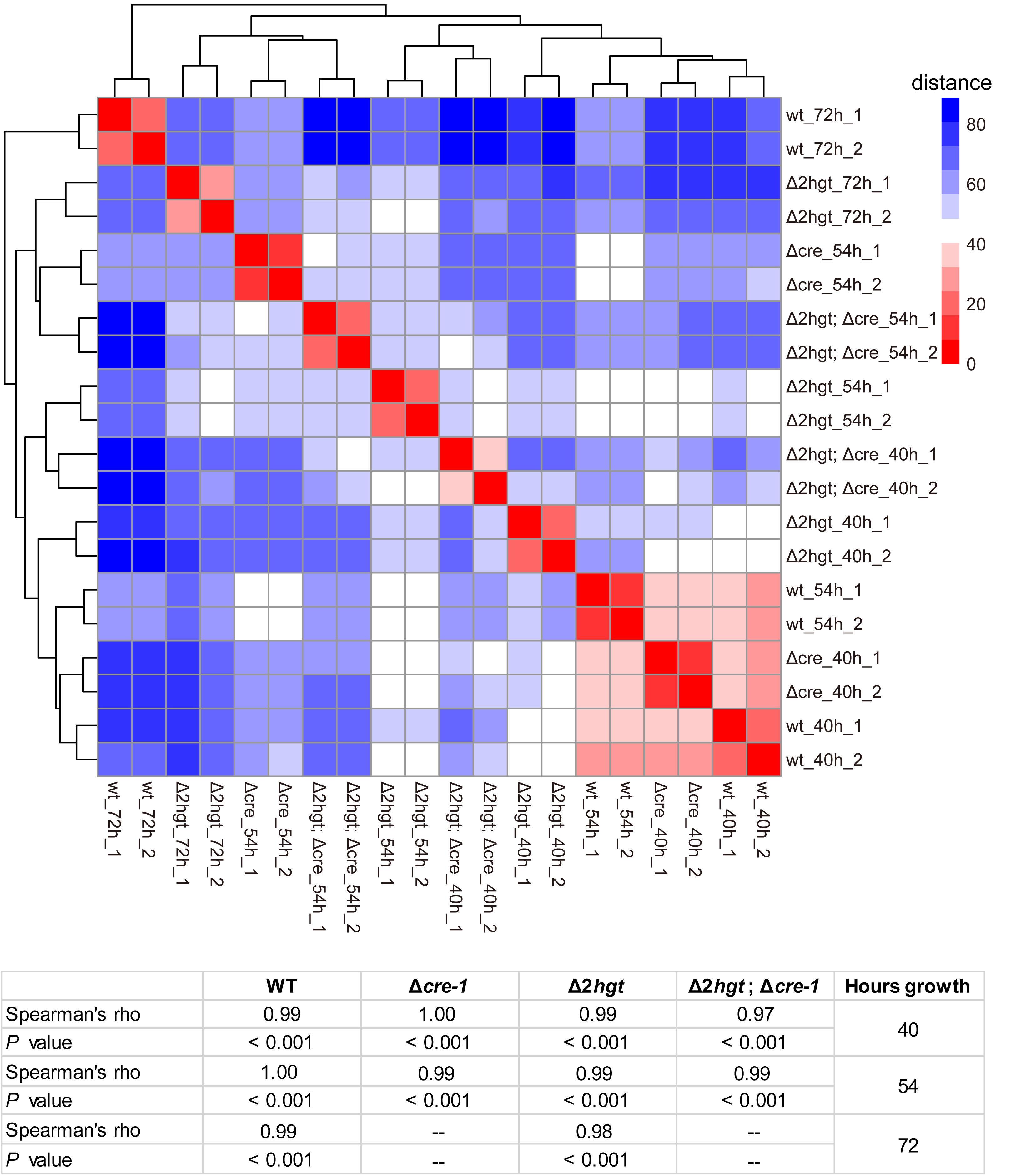

Supplement: Supplementary file 9 — Additional file 9: Figure S6. Validation of RNA-Seq data of the WT, Δcre-1, Δ2hgt, and Δ2hgt;Δcre-1 grown on Avicel for the indicated times. The results of sample-to-sample clustering and Spearman analysis are shown. [file 13068_2017_705_MOESM9_ESM.tiff]

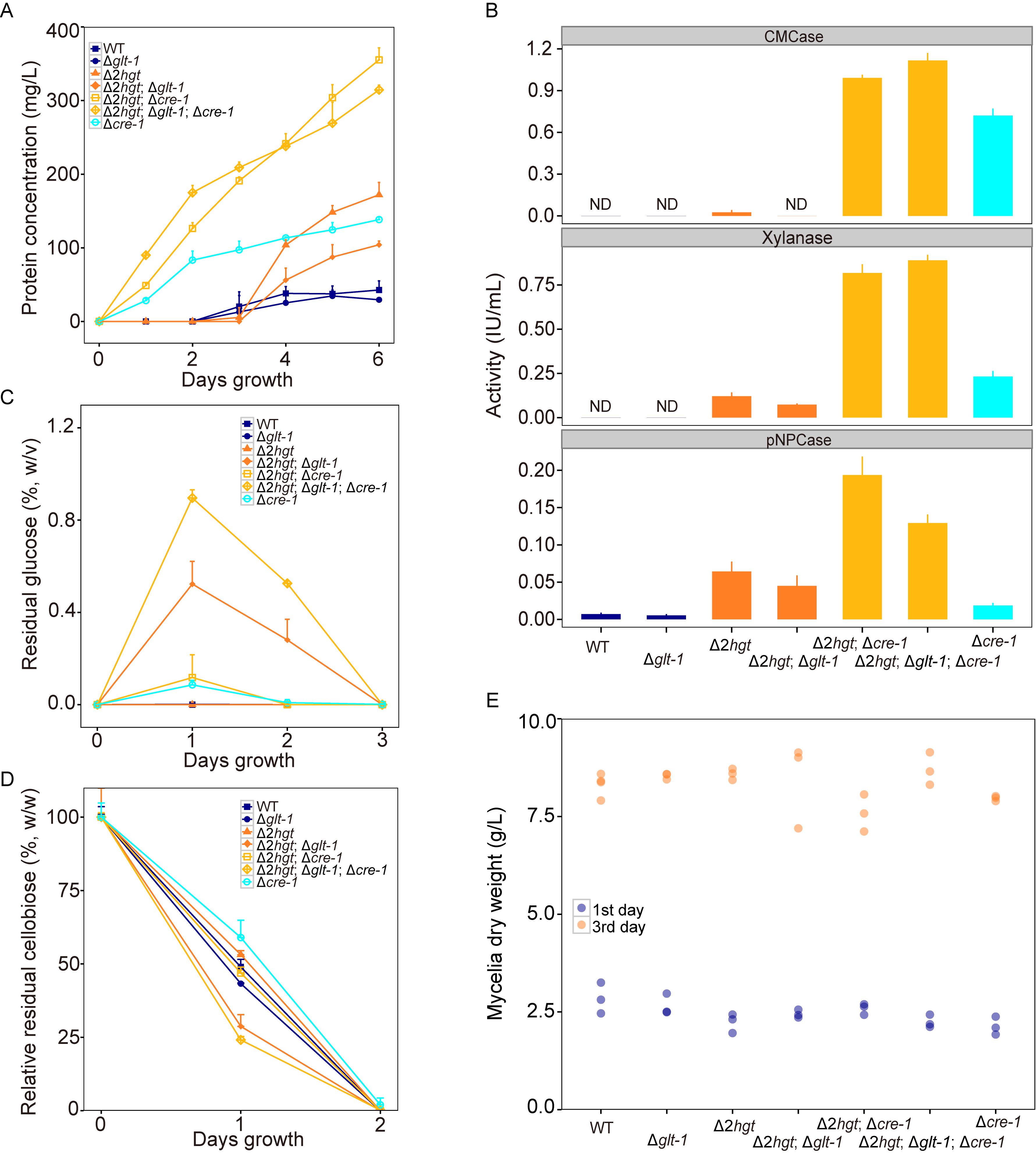

Supplement: Supplementary file 12 — Additional file 12: Figure S7. Phenotypes of WT, Δglt-1, Δcre-1, Δ2hgt, Δ2hgt;Δglt-1, Δ2hgt;Δcre-1, and Δ2hgt;Δglt-1;Δcre-1 strains grown on 2.0% cellobiose as the sole carbon source. (A) Dynamics of secreted protein production by the tested strains. (B) Endo-glucanase (CMCase), xylanase, and exo-glucanase (pNPCase) of 7-day-culture supernatants. (C) Residual glucose and (D) cellobiose of the supernatants. (E) Dried mycelial weights of tested strains grown for 1 and 3 days. Values represent averages of triplicates; error bars show standard deviations. [file 13068_2017_705_MOESM12_ESM.tiff]

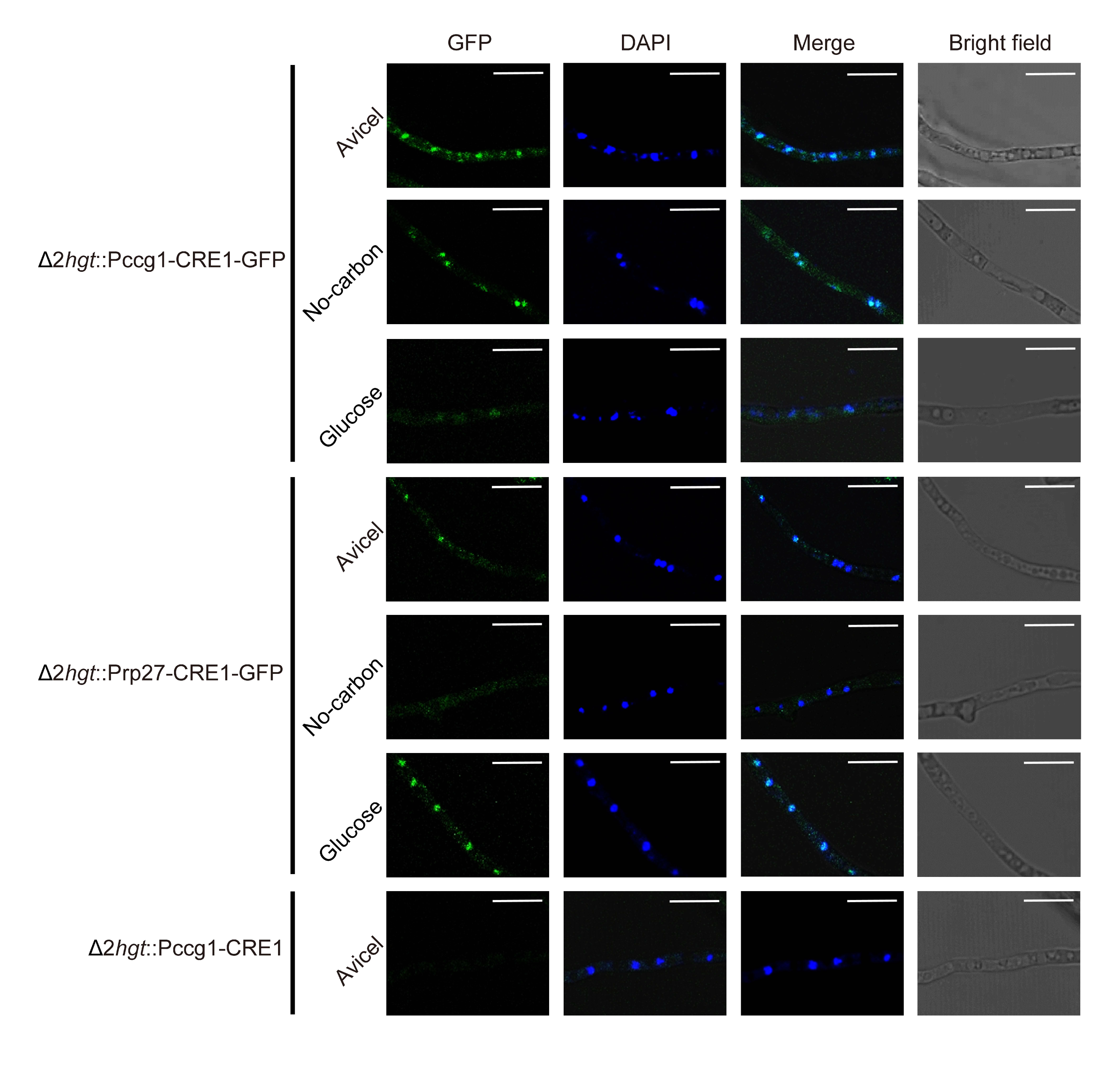

Supplement: Supplementary file 13 — Additional file 13: Figure S8. Confocal imaging of the Δ2hgt mutant expressing CRE-1 controlled by ccg-1 or ribosomal protein 27 promoters after 16 h of mycelial pre-growth under no-carbon, Avicel, and glucose conditions, respectively. The scale bar corresponds to 10 µm. [file 13068_2017_705_MOESM13_ESM.tiff]

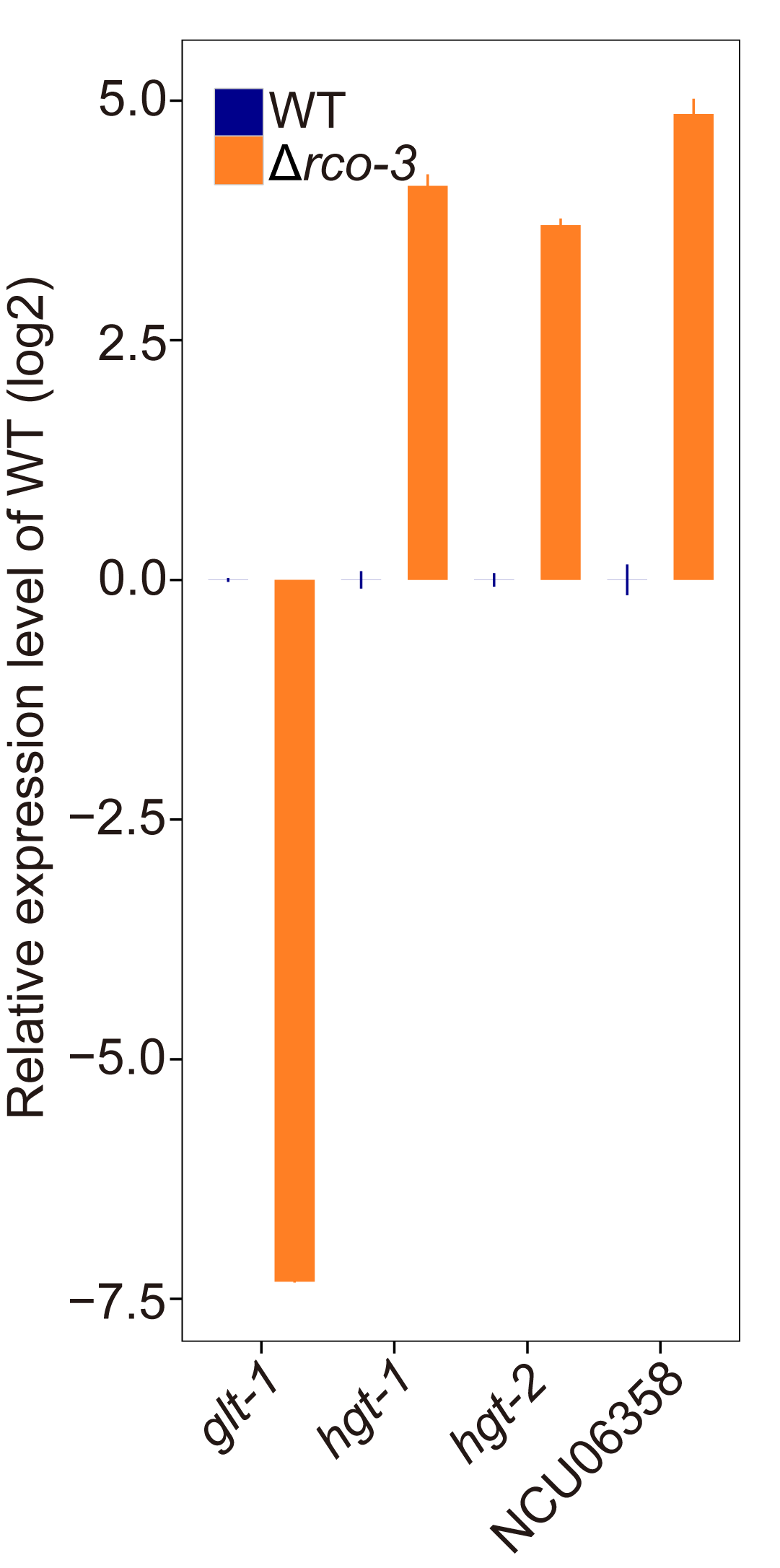

Supplement: Supplementary file 14 — Additional file 14: Figure S9. Relative expression levels of glt-1, hgt-1/-2, and another putative glucose transporter, NCU06358, in the Δrco-3 mutant vs. the WT treated with 0.5% glucose for 1 h. Average values and standard deviations of two biological replicates/two technical replicates are shown. [file 13068_2017_705_MOESM14_ESM.tiff]
